# International Study of Childhood Obesity, Lifestyle and the Environment (ISCOLE): Contributions to Understanding the Global Obesity Epidemic

**DOI:** 10.3390/nu11040848

**Published:** 2019-04-15

**Authors:** Peter T. Katzmarzyk, Jean-Philippe Chaput, Mikael Fogelholm, Gang Hu, Carol Maher, Jose Maia, Timothy Olds, Olga L. Sarmiento, Martyn Standage, Mark S. Tremblay, Catrine Tudor-Locke

**Affiliations:** 1 Pennington Biomedical Research Center, 6400 Perkins Road, Baton Rouge, LA 70808, USA; gang.hu@pbrc.edu; 2 Children’s Hospital of Eastern Ontario Research Institute, Ottawa, ON K1H 8L1, Canada; jpchaput@cheo.on.ca (J.-P.C.); mtremblay@cheo.on.ca (M.S.T.); 3 Department of Food and Environmental Sciences, University of Helsinki, 00014 Helsinki, Finland; mikael.fogelholm@helsinki.fi; 4 School of Health Sciences, Sansom Institute, University of South Australia, Adelaide, SA 5001, Australia; Carol.Maher@unisa.edu.au (C.M.); timothy.olds@unisa.edu.au (T.O.); 5 Faculdade de Desporto, University of Porto, Rua Dr. Plácido Costa, 91, 4200-450 Porto, Portugal; jmaia@fade.up.pt; 6 School of Medicine, Universidad de los Andes, Bogota 11001000, Colombia; osarmien@uniandes.edu.co; 7 Department for Health, University of Bath, Bath BA2 7AY, UK; m.standage@bath.ac.uk; 8 Department of Kinesiology, University of Massachusetts, Amherst, MA 01003, USA; ctudorlocke@umass.edu

**Keywords:** pediatric, overweight, epidemiological transition, collaboration

## Abstract

The purpose of this review is to summarize the scientific contributions of the International Study of Childhood Obesity, Lifestyle and the Environment (ISCOLE) in extending our understanding about obesity in children from around the world. ISCOLE was a multi-national study of 9 to 11 year-old children from sites in 12 countries from all inhabited continents. The primary purpose was to investigate relationships between lifestyle behaviors and obesity, and the influence of higher-order characteristics such as behavioral settings, and physical, social and policy environments. ISCOLE has made several advances in scientific methodology related to the assessment of physical activity, dietary behavior, sleep and the neighborhood and school environments. Furthermore, ISCOLE has provided important evidence on (1) epidemiological transitions in obesity and related behaviors, (2) correlates of obesity and lifestyle behaviors at the individual, neighborhood and school levels, and (3) 24-h movement behaviors in relation to novel analytical techniques. A key feature of ISCOLE was the development of a platform for international training, data entry, and data quality for multi-country studies. Finally, ISCOLE represents a transparent model for future public-private research partnerships across low, middle and high-income countries.

## 1. Introduction

The prevalence of childhood obesity has increased significantly in recent years and remains high in many countries [1]. Given the global nature of the problem, a greater understanding of context-specific correlates of obesity is required in order to develop effective interventions that can be translated from one setting to another. To date, there have been only limited attempts to understand the correlates of adiposity or obesity in specific world regions using standardized methods. Large multi-country studies of childhood obesity and/or related behavioral risk factors (physical activity, diet, etc.) have been largely limited to the European region [2,3,4,5,6]. Therefore, the primary aim of the International Study of Childhood Obesity, Lifestyle and the Environment (ISCOLE) was to investigate relationships between lifestyle behaviors and obesity, and the influence of higher-order characteristics such as behavioral settings, and the physical, social and policy environments, on the observed relationships within and between countries that vary widely in levels of human development [7]. ISCOLE was a multi-national study of 9–11 year-old children from research sites in 12 countries from all inhabited continents ranging widely in environmental and socio-cultural contexts. 

The purpose of this paper is to summarize the scientific contributions of ISCOLE in extending our understanding about obesity in children from around the world. The focus is primarily on the results from analyses that utilized the 12-country dataset; nevertheless, a large number of papers have also been generated using country-specific datasets or data from small clusters of countries. A complete list of scientific peer-reviewed papers to date from ISCOLE can be found in the online Supplementary Materials (Supplementary File S1). 

## 2. Study Design

A detailed description of the ISCOLE design and methods has been published elsewhere [7]. Briefly, ISCOLE was a multi-national, cross-sectional study conducted in 12 countries (Australia, Brazil, Canada, China, Colombia, Finland, India, Kenya, Portugal, South Africa, United Kingdom, United States) from all inhabited continents. A total of 7372 9–11 year old children participated in ISCOLE [8]. In addition to including sites from countries across a wide range of human development, children were sampled across a range of family socio-economic status within each country. By design, the ISCOLE samples are not representative of the populations from which the participants were drawn. However, an analysis of ISCOLE data compared to other available studies across many world regions suggests that there is no evidence that the ISCOLE samples are systematically biased [9]. These results suggest that ISCOLE data could be used cautiously to inform the development of country-level interventions when other data are lacking. 

Table 1 provides descriptive characteristics of the sample by study site, ranked according to the prevalence of obesity. The Human Development Index (HDI) of the study sites ranged from 0.509 in Kenya to 0.929 in Australia. The average age of the sample was 10.4 years, and the prevalence of obesity ranged from 5.4% in Finland to 23.7% in China. Level of parental education also varied among the study sites; with the proportion of the sample with parents having at least a bachelor’s degree ranging from 12.9% in South Africa to 73.4% in India.

All ISCOLE data were collected under a standardized research protocol using the same instrumentation at all study sites. Data included objectively measured indicators of adiposity and obesity (body mass index (BMI), waist circumference, body fat), lifestyle behaviors related to obesity (diet, physical activity, sleep, etc.), demographics and family health history, the home and neighborhood environment, and the school environment. All information was entered remotely (anthropometry, questionnaires, etc.) or uploaded (accelerometry) on a secure web-based data entry platform. ISCOLE employed a rigorous quality assurance and quality control program. This program included comprehensive in-person training and certification of all investigators and staff, random remote source data verification, in-person site monitoring visits, and data cleaning and final source data verification [7].

## 3. Advances in Scientific Methodology Related to the Assessment of Physical Activity, Sleep, Dietary Behavior, and the Neighborhood, Home and School Environments

Mounting a multi-national study of the scale of ISCOLE required the development of novel methods and the adaptation of existing tools that could be applied in sites that ranged considerably in level of human development. This section summarizes some of the methodological advances that were developed during the planning and implementation of ISCOLE. As described above, we have made all of our protocols and algorithms publicly available, and we have summarized our contributions to the use of accelerometry in large studies in detail elsewhere [11,12].

### 3.1. Physical Activity

A major strength of ISCOLE was the objective assessment of physical activity and sedentary behavior using a waist-mounted accelerometer protocol that was deployed in all study sites [7,11]. Awake-time wear protocols typically require the participants to remove their accelerometer before going to bed and then to reapply it in the morning upon waking. Concerns about wear time compliance have led some investigators to adopt a wrist-mounted rather than a waist-mounted protocol [13]. For example, the U.S. National Health and Nutrition Examination Survey (NHANES) switched from a waist-mounted to a wrist-mounted protocol between the 2005–2006 and 2011–2012 cycles of the survey [13]. In ISCOLE, we chose to attempt to improve wear time compliance by using a waist-mounted 24-h protocol rather than moving to a wrist-mounted protocol [7,14].

The 24-h protocol employed in ISCOLE resulted in impressive increases in wear time in comparison to previous studies. The average wear time in ISCOLE was 22.8 h per day [8]. Given that NHANES used a wake-only protocol, no direct comparisons can be made for total wear time. However, we conducted a study comparing the US ISCOLE site with the 2003–2006 NHANES (that used the waist mounted protocol), and the awake wear time in ISCOLE was 14.7 h per day compared to 13.7 h per day in NHANES, which represents a one hour per day improvement when using the 24-h protocol [14].

Using a 7-day protocol allowed us to estimate the reliability of accelerometer-determined physical activity and sedentary behavior [15]. The estimated minimum number of days needed to achieve a reliability of G ≥ 0.8 ranged from 5 to 9 for boys and 3 to 11 for girls for light physical activity; 5 to 9 and 3 to 10 for moderate-to-vigorous intensity physical activity; 5 to 10 and 4 to 10 for total activity counts; and 7 to 11 and 6 to 11 for sedentary time, respectively [15]. The results demonstrate that, in most cases, close to seven days of monitored time is required to achieve adequate reliability; and future studies should take this into account when designing their protocols.

### 3.2. Sleep

The availability of seven days of 24-h accelerometry data in ISCOLE provided an opportunity to develop algorithms to objectively identify the sleep period [16,17]. Over several months, we developed a fully automated algorithm for identifying the nocturnal total sleep episode time in two stages. The first step was to develop and validate an initial algorithm against expert visual inspection of the data [16]. The initial algorithm combined aspects of the Sadeh algorithm [18] for sleep–wake scoring, made use of the inclinometer function in the accelerometer, and built upon the framework of the publicly available non-wear algorithm developed by the National Cancer Institute [19]. The initial algorithm identified sleep onset (i.e., ‘bedtime’) and sleep offset (i.e., ‘waking’) times. The second step was to refine the algorithm by adding the ability to identify disrupted nocturnal sleep episodes (and exclude episodes of nighttime non-wear/wakefulness) and avoid misclassification of daytime non-wear or sedentary behavior as sleep [17]. Compared with sleep logs, we achieved acceptable levels of accuracy (<10% mean absolute percent difference) [17]. The Pennington Biomedical Research Center (PBRC) hosts public web-based access to both the original [20] and refined [21] algorithms. As a companion to the sleep algorithms, we have published a full catalog of nocturnal sleep-related variables in ISCOLE [22].

### 3.3. Dietary Behavior

The primary dietary information used in ISCOLE was collected using a Food Frequency Questionnaire (FFQ) adapted from the Health Behavior in School-aged Children Survey [23]. The ISCOLE FFQ asks about the consumption of 23 food items, and was adapted for use in each of the 12 study sites [7]. We conducted a reliability and validity study in three culturally different study sites (Finland, US, and Colombia) [24]. Reliability correlation coefficients from two surveys completed ~5 weeks apart ranged from 0.37 to 0.78 and gross misclassification for all food groups was <5%. Validity correlation coefficients were below 0.5 for 22/23 food groups and gross misclassification was <5% for 22/23 food groups. Over- or underestimation did not appear for 19/23 food groups [24].

**Figure 1:**
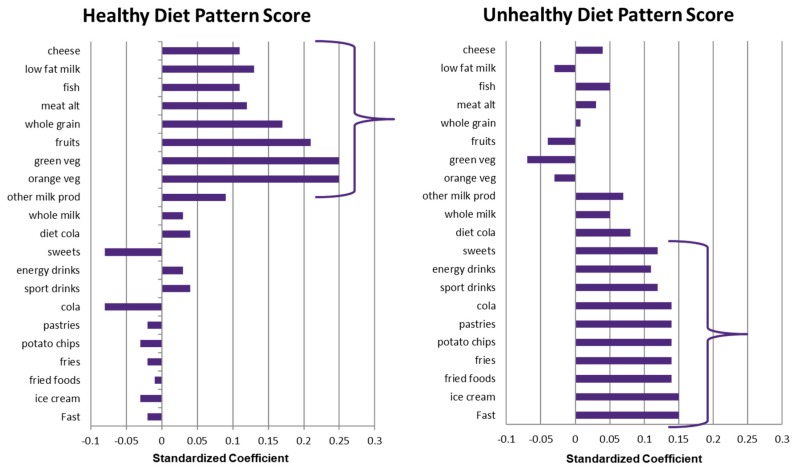
Principal component loadings for the healthy and unhealthy diet pattern scores in the International Study of Childhood Obesity, Lifestyle and the Environment (ISCOLE) (all sites combined), from Mikkila et al. [25].

To identify dietary patterns, principal components analyses (PCA) were carried out using weekly portions as input variables [25]. Both site-specific and pooled data showed that dietary behaviors in ISCOLE to be well defined by two component solutions. We labelled the first component as the “unhealthy diet pattern”, which included sugar-sweetened sodas, fast foods, ice cream, fried food, French fries, potato chips, and cakes. The second component we characterized as the “healthy diet pattern”, which included dark-green vegetables, orange vegetables, fish, cheese, whole grains and fruits. Figure 1 presents the loadings for the two principal components.

### 3.4. Neighborhood, Home and School Environments

Information on several aspects of the neighborhood, home and school environments were collected in ISCOLE using a variety of approaches. A neighborhood and home environment questionnaire, which was based on the Neighborhood Impact on Kids (NIK) survey [26], was completed by parents/guardians. The school environment was assessed using two approaches. First, a school administrator questionnaire, which covered school facilities, healthy eating and physical activity policies, extracurricular activities, frequency of physical education and breaks (recess), and availability of healthy and unhealthy food, was completed by a school official [7]. Second, a school audit of the physical environment was performed at each participating school by one of the study staff. Each site completed a reliability audit (simultaneous audits by two independent, certified data collectors) for a minimum of two schools or at least 5% of their school sample [27]. For the assessed environmental features, inter-rater reliability (kappa) ranged from 0.37 to 0.96; 18 items (42%) were assessed with almost perfect reliability (Κ = 0.80–0.96), and a further 24 items (56%) were assessed with substantial reliability (Κ = 0.61–0.79) [27]. These results suggest that the ISCOLE school audit can be used to conduct reliable objective audits of the school environment across diverse, international school settings. However, the administration of the school audit tool can be challenging in some contexts, such as in countries where snow may cover or change aspects of the school environment. Furthermore, research is required to validate these tools under different environmental conditions.

## 4. Epidemiological Transitions in Obesity and Related Behaviors

The theory of epidemiologic transition characterizes long-term changes in patterns of morbidity and mortality away from causes related to undernutrition and infectious diseases towards chronic ‘man-made’ diseases as countries become more developed [28]. Related to the concept of epidemiological transition, theories about parallel nutritional and physical activity transitions have been described [29,30]. The nutrition transition is characterized by a shift away from traditional diets that were based on staple grains, local legumes, and fruits and vegetables, towards a diet comprised of more animal-based food products and processed food high in saturated fats and sugar [29]. The physical activity transition is characterized by long-term shifts in physical activity patterns away from necessity (acquiring food, water and shelter, escaping predation, procreation or transport in settings with low motor vehicle availability) towards a largely inactive lifestyle in high-income countries where physical activity has been successfully engineered out of our everyday lives. In high-income countries, humans no longer need to be physically active out of necessity but instead act out of choice to be physically active because of enjoyment, maintenance of body weight, employment, and the prevention of chronic diseases [30]. Whereas in lower-middle income countries where car availability remains relatively low in comparison to high income countries, physical activity could be more reflective of purposeful transport rather than leisure pursuits. These parallel transitions in nutrition and physical activity may contribute to the increased chronic disease burden associated with the epidemiological transition, such as higher rates of obesity, type 2 diabetes, cardiovascular disease, and many cancers.

**Figure 2:**
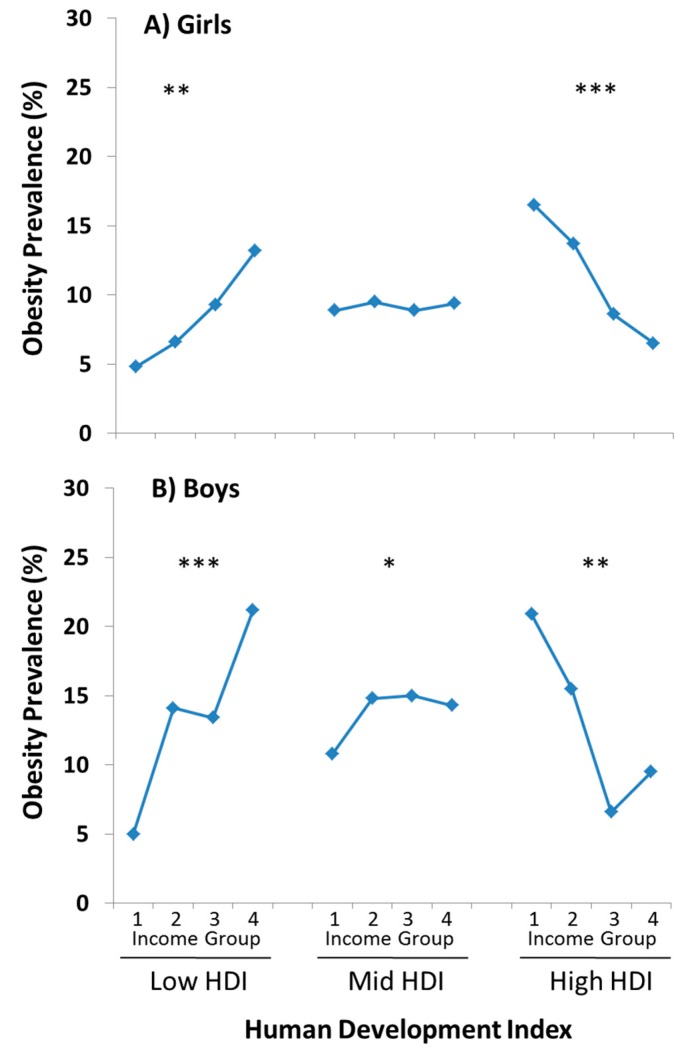
Income gradients in obesity prevalence across levels of HDI in (**A**) girls and (**B**) boys from ISCOLE. Low, middle and high human development index (HDI) correspond to the 10th, 50th and 90th percentiles of the ISCOLE sample (HDI = 0.52, 0.72 and 0.91, respectively). Tests for linear trend are indicated: * *p* < 0.05; ** *p* < 0.001; *** *p* < 0.0001. Figure is adapted from Broyles et al. [32].

A review of studies from Western developed countries published between 1990 and 2005 concluded that there was a significant inverse association between socio-economic status and obesity and that positive associations had all but disappeared [31]. However, little is known about how indicators of childhood obesity vary across levels of socio-economic status among countries at different levels of the human development index (HDI). ISCOLE was uniquely positioned to answer this question. Our results demonstrated that BMI and percent body fat were positively associated with family income in countries with low HDI, negatively associated in countries at high HDI, with no association in countries with an HDI in the midrange [32]. Similar patterns were observed for the association between HDI and the prevalence of obesity (Figure 2), reflecting variability in the stages of nutrition and physical activity transitions among countries.

In addition to obesity, we tested for socio-economic gradients in physical activity, dietary patterns and sleep duration in ISCOLE [33,34,35]. In girls, time spent in moderate-to-vigorous physical activity was negatively associated with family income at the 10th and 50th percentiles HDI (all *p* < 0.012); and positively related with family income at the 90th percentile (*p* = 0.044) [35]. In boys, time spent in moderate-to-vigorous physical activity was also negatively associated with family income at the 10th and 50th percentiles of HDI (both *p* < 0.001) [35]. These results are consistent with the existence of a physical activity transition. A parallel analysis of dietary patterns demonstrated that lower family income was associated with a higher “unhealthy” dietary pattern score and a lower “healthy” dietary pattern score in many countries; however, the pattern was not reflective of a nutrition transition in dietary patterns in these counties [34]. Finally, we also explored the association between sleep duration and family income [33]. No significant associations were observed in any site, and the summary odds ratio was also not significant (OR = 0.94; 95% Confidence Interval (CI) = 0.60 – 1.47) [33].

In summary, in ISCOLE, we found evidence of epidemiological transitions in obesity and physical activity, but not for dietary patterns and sleep duration. Furthermore, research is required to better characterize country-level changes in these behaviors using temporal surveillance data in countries undergoing rapid economic and social development.

## 5. Correlates of Obesity and Lifestyle Behaviors at Multiple Levels

### 5.1. The Socio-Ecological Model

The socio-ecological model is a variant of the root Ecological Systems Theory firstly developed by Bronfenbrenner [36]. Socio-ecological models have been proposed as frameworks to think about the influences of factors at several levels (individual, social, physical and policy environments) on obesity, physical activity and dietary intake [37,38,39]. ISCOLE was designed to help answer some questions about the contributions of factors at multiple levels of the socio-ecological model to childhood obesity and related behaviors. Our study was a nested design, with individuals (Level 1) nested within schools (Level 2), which were in turn nested within study sites (Level 3). This design necessitated the use of multi-level mixed models for data analysis. This approach also allowed us to partition the percentage of the variance in several variables that is explained by factors at all three levels. The multilevel model is highly elegant in its statistical formulation [40], as well as its versatility [41] which made it a valid model to use with the hierarchical system of information gathered from different levels as in ISCOLE—individuals nested within schools which are nested within research sites. Furthermore, within-level as well as cross-level interactions were considered as per the ISCOLE framework, and these were modeled and statistically tested to verify their substantive tenability.

We undertook an analysis that estimated the proportion of the variance in several key variables at the study site, school and individual levels [42]. The proportion of the variance in BMI and waist circumference explained at the individual level is greater than 90%; the proportion of the variance explained in dietary patterns, sleep, physical activity and sedentary time at the individual level ranges between 66% and 88%, while the proportion of the variance in in-school physical activity and in-school sedentary behavior explained at the site and school levels is between 46% and 75%, with less of a contribution from individual-level factors [42]. These results suggest that interventions that target policy and environmental changes for increasing school-based physical activity and reducing in-school sedentary behavior may enhance obesity intervention efforts.

### 5.2. Obesity

Several analyses have been undertaken to identify the correlates of obesity in ISCOLE. Our first investigation involved determining the associations between several lifestyle traits (healthy diet patterns, unhealthy diet patterns, moderate-to-vigorous physical activity, TV viewing time, and sleep duration) and the presence of obesity [8]. The odds ratios for obesity (per standard deviation of the predictor variable) were 0.51 (95% CI: 0.45–0.57) for moderate-to-vigorous physical activity, 0.79 (95% CI: 0.72–0.86) for sleep duration, and 1.11 (95% CI: 1.04–1.19) for TV viewing time, while the diet pattern scores were not related to obesity [8]. The results were consistent in boys and girls. These findings led to an in-depth examination of the associations among obesity and accelerometer-derived measures of physical activity and sedentary behavior [43]. In the overall sample, the odds ratios for obesity (per standard deviation of each predictor variable) were significant for sedentary time (1.19; 1.08–1.30), moderate-to-vigorous physical activity (0.49; 95% CI, 0.44–0.55), and vigorous physical activity (0.41; 0.37–0.46). Furthermore, the associations of moderate-to-vigorous physical activity and vigorous physical activity with obesity were significant in all 12 sites, whereas the association between sedentary time and obesity was significant in five of the 12 sites [43].

Active school transport is one potential opportunity for children to accumulate physical activity during the day. Thus, we examined the association between active school transport and indicators of adiposity in ISCOLE [44]. After adjusting for several covariates, children who reported active school transport were less likely to be obese (odds ratio = 0.72, 95% CI: 0.60–0.87) and had a lower BMI z-score, percent body fat and waist circumference (all *p* < 0.05) compared with those who reported motorized travel [44]. Furthermore, the associations between active school transport and obesity did not differ by country or by sex.

Although we found that dietary patterns were not related to obesity in ISCOLE [8], we further explored the association between specific dietary behaviors and obesity. For example, frequent breakfast consumption was associated with lower BMI z-scores compared with occasional (*p* < 0.0001) and rare (*p* < 0.0001) consumption, as well as lower percentage body fat compared with occasional (*p* < 0.0001) and rare (*p* < 0.0001) consumption [45]. These associations differed significantly across study sites, and further research is required to understand these differences. We also explored the association between soft drink consumption and obesity [46]. There was a significant linear trend for increasing BMI z-scores across increasing consumption of regular soft drinks in boys (*p* = 0.049), but not in girls. On the other hand, there was no significant linear trend across categories of diet soft drink consumption in boys, but there was a graded, positive association in girls for BMI z-score (*p* = 0.0002) [46].

Evidence from high-income countries has identified associations between gestational diabetes and birth weight with subsequent childhood obesity. We explored these associations in the multi-national sample from ISCOLE. Compared to children with mothers who did not experience gestational diabetes, children with mothers who experienced gestational diabetes had an odds ratio of 1.53 (95% CI: 1.03–2.27) for obesity, 1.73 (95% CI: 1.14–2.62) for central obesity, and 1.42 (95% CI: 0.90–2.26) for high percentage body fat [47]. Furthermore, the odds ratios for obesity were 1.45 (95% CI: 1.10–1.92) for those with birthweight of 3500–3999 g and 2.08 (95% CI: 1.47–2.93) for those with birthweight ≥4000 g, compared with those with birthweight of 2500–2999 g [48]. The positive association between birth weight and obesity was linear in girls, whereas it was U-shaped in boys. We further explored the association between birthweight and obesity by examining interactions with physical activity and sedentary behavior. Interestingly, the positive association between birthweight and obesity was significant among children with either low moderate-to-vigorous physical activity or high sedentary time, but not among children with either high moderate-to-vigorous physical activity or low sedentary time [49].

Building upon the evidence supporting the inter-generational transmission of obesity, we found that parental overweight was associated with childhood overweight in the overall ISCOLE sample [50]. Furthermore, parental education was differentially associated with childhood overweight across the ISCOLE study sites, and more research is required to understand the context-specific associations between parental education and childhood overweight and obesity.

### 5.3. Physical Activity and Sedentary Behavior

Current public health recommendations call for children and youth to accumulate at least 60 min of moderate-to-vigorous physical activity every day [51]. In ISCOLE, 4.8% of children achieved ≥60 min of moderate-to-vigorous physical activity for all seven days of the week, while 25.5% attained the recommendation ≥5 days [52]. Furthermore, a total of 18.8% of the sample did not accumulate ≥60 min of moderate-to-vigorous on any of the monitored days [52]. There was variability in compliance to the guidelines across sites: the mean number of days of compliance ranged from 1.8 days per week in the United States to 3.5 days per week in Colombia [52]. Given the availability of 24-h, time-stamped accelerometry data and detailed information about the start and stop times for each student’s school in ISCOLE, we were able to differentiate between before-school, during-school and after-school physical activity and sedentary behavior.

Physical education classes are an important opportunity for physical activity in children and youth. In ISCOLE, approximately 25% of participants reported attending physical education classes on three or more days per week [53]. After adjusting for several covariates, children who took physical education classes were more likely to have higher levels of physical activity and shorter time in spent in sedentary behavior both in and out of school during the school week [53].

We also found that children who used active school transportation had significantly higher weekday moderate-to-vigorous physical activity and significantly lower light physical activity before school compared with children who used motorized transport to school [54]. On average, children who used active transportation accumulated 6.0 (95% CI: 4.7–7.3) min more moderate-to-vigorous physical activity per day than children who used motorized transportation. There was wide variability in the prevalence of active school transportation across study sites, which varied from 5.2% in India to 79.4% in Finland [55]. We found wide variability in the correlates that were associated with active school transport across study sites. Longer trip duration (≥16 min vs. ≤15 min) was associated with lower odds of active school transportation in eight sites; whereas individual and neighborhood factors were associated with active school transportation in three sites or less [55].

In ISCOLE, we also investigated home and neighborhood correlates of physical activity. Across sites, children with at least one piece of electronic media in their bedroom had lower levels of moderate-to-vigorous physical activity than those who did not (*p* < 0.001) [56]. More frequent physical activity in the home and yard, ownership of more frequently used play equipment, and higher social support for physical activity were also associated with higher moderate-to-vigorous physical activity (*p* < 0.0001). However, association between play equipment ownership and moderate-to-vigorous physical activity varied across study sites (*p*_interaction_ < 0.01), suggesting that cultural differences should be studied further when developing interventions or making recommendations [56].

Aspects of the neighborhood social environment (collective efficacy and perceived crime) were also studied in ISCOLE as potential correlates of moderate-to-vigorous physical activity [57]. Collective efficacy was inversely associated with moderate-to-vigorous physical activity among children in low/lower-middle-income countries (β = −1.96; 95% CI: −3.72, −0.19) while it was positively associated with moderate-to-vigorous physical activity among children in high-income countries (β = 1.86; 95% CI: 0.76, 2.96) [57]. Perceived crime was significantly associated with lower moderate-to-vigorous physical activity (β = −2.12; 95% CI: −3.18, −1.06) among children in high-income countries but was not significantly associated with moderate-to-vigorous physical activity among children from low/lower-middle-income countries or upper-middle-income countries [57]. These results demonstrate heterogeneity in associations between aspects of the neighborhood environment and physical activity that need to be taken into account when developing strategies that target these correlates in different settings.

In addition to physical activity, we also investigated associations of 21 potential correlates with accelerometer-determined sedentary time and self-reported TV viewing time [58]. Boys reported greater TV viewing time than girls, while in 9 of 12 sites, girls engaged in more objectively-measured sedentary time than boys. Common correlates of sedentary time and TV viewing time included excess weight status, not meeting physical activity recommendations, and having a TV in the bedroom [58].

The associations between sleep and movement behaviors are currently of great interest [59]. In ISCOLE, sleep duration was negatively associated with moderate-to-vigorous physical activity and sedentary time, while sleep efficiency was negatively related to moderate-to-vigorous physical activity and positively associated with sedentary time [60]. The availability of time-stamped accelerometry data allowed us to examine temporal associations between sleep, physical activity and sedentary time [61]. Results showed that the relationships between sleep and physical activity and sedentary time are bi-directional. For example, for each one standard deviation (SD) unit increase in sleep duration, sedentary behavior was 0.04 SD units lower the following day, while light physical activity and moderate-to-vigorous physical activity were 0.04 and 0.02 SD units higher, respectively. Sleep duration was 0.02 SD units lower and 0.04 SD units higher for each one SD unit increase in sedentary time and moderate-to-vigorous physical activity, respectively [61]. While these results highlight the interactions between sleep and movement behaviors, the small effect sizes suggest that the clinical implications may be modest.

### 5.4. Dietary Patterns

**Figure 3:**
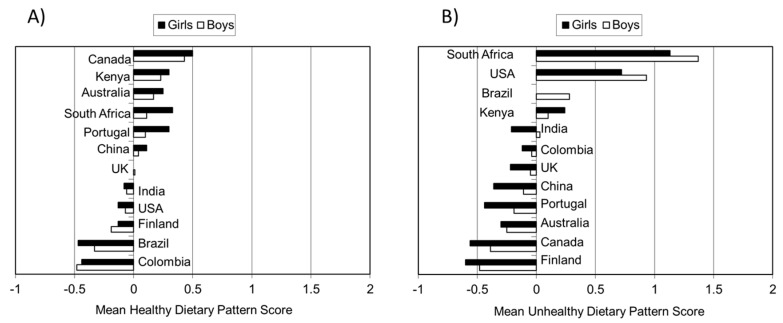
Mean dietary pattern scores across ISCOLE study sites. (**A**) presents the mean scores for the “healthy dietary pattern” and (**B**) presents the mean scores for the “unhealthy dietary pattern”. Data were obtained from Mikkila et al. [25].

As previously described, “healthy” and “unhealthy” dietary pattern scores were derived from FFQ data in ISCOLE [25]. Figure 3 presents the mean dietary scores across study sites. The results demonstrate variability in dietary pattern scores across countries, with the highest healthy dietary pattern score found in Canada, while the lowest is found in Colombia. Finland had the lowest unhealthy dietary pattern score while South Africa had the highest.

We explored the potential home and school environments as correlates of dietary patterns [62]. Here, we found that more meals eaten outside home and school were associated with higher unhealthy diet pattern scores. Furthermore, low availability of empty-calorie foods at home was found to be more important than high availability of wholesome foods at keeping unhealthy diet pattern scores low. The availability of wholesome foods at home was positively associated with the healthy diet pattern scores, while food availability at school was not associated with the dietary patterns [62]. In the ISCOLE sample, the home food environment was more significant than the school food environment in predicting the child’s dietary patterns.

Given the availability of objective measures of sleep in ISCOLE, coupled with the FFQ data, we were able to study the association between sleep and dietary variables [60,63]. Both sleep duration and sleep efficiency were negatively associated with the unhealthy diet pattern score [60]. Interestingly, shorter sleep duration was associated with higher intake of regular soft drinks, while earlier bedtimes were associated with lower intake of regular soft drinks and higher intake of energy drinks and sports drinks [63]. More research is required to better understand the underlying mechanisms that might link beverage consumption to sleep patterns.

### 5.5. Higher-Order Correlates

Given that ISCOLE data were collected across multiple seasons in several geographical regions of the world, we were able to explore associations with other potential “higher-order” correlates of obesity and lifestyle behaviors. For example, we explored the association between moon phase, sleep and physical activity using data from 33,710 24-h accelerometer recordings of sleep and activity [64]. While differences in moderate-to-vigorous physical activity, light physical activity, and sedentary time between moon phases were negligible and non-significant (<2 min/day), sleep duration was significantly shorter (~5 min/night) during the full moon phase compared to the new moon phase [64]. Despite the statistical significance of the association between moon phase and sleep duration, the magnitude of the difference is unlikely to be clinically important.

The associations of weather with physical activity and sedentary time were explored using data from the Australian and Canadian ISCOLE sites [65]. Daily maximal temperature was significantly associated with physical activity and sedentary time in both Australia and Canada, and daily rainfall was negatively associated with physical activity in Australia and positively associated with sedentary time in Canada [65]. The results from both countries indicated that the best levels of physical activity and sedentary time occurred in a range between 20° and 25° Celsius. These results highlight the importance of taking weather into account in the development of intervention and surveillance strategies related to physical activity and sedentary behavior.

The associations of outdoor time with BMI, physical activity, sedentary time and dietary patterns were also explored in ISCOLE [66,67]. Time spent outside and dietary patterns were assessed by questionnaire, while time spent in physical activity and sedentary behavior were determined by accelerometry [7]. Time spent outside was not associated with BMI z-scores; however, each additional hour per day spent outdoors was associated with higher moderate-to-vigorous and light physical activity, and lower sedentary time [67]. Effect sizes were generally weaker in lower-middle-income countries. However, the evaluation of the Ciclovia in Colombia to promote physical activity outside on the streets was associated with higher moderate-to-vigorous and light physical activity, and lower sedentary time on Sundays [68]. Furthermore, time spent outdoors was positively associated with higher healthy dietary pattern scores, but there was no association with the unhealthy dietary pattern scores [66]. Similar patterns were observed in boys and girls, and across study sites. Research using longitudinal study designs is required to elucidate the mechanisms behind these observed associations.

## 6. The 24-h Movement Behavior Cycle in Relation to Integrated Guidelines and Novel Analytical Techniques

The focus of physical activity guidelines in public health has typically been on the promotion of moderate-to-vigorous physical activity. However, there has been increasing interest in understanding the health impacts associated with multiple movement behaviors (i.e., sedentary behavior, light activity, moderate-to-vigorous activity, and sleep) [59]. Canada has recently released *24-h Movement Guidelines for Children and Youth*, which attempts to integrate these movement behaviors [69]. For example, the guidelines recommend that 10 year-old children should accumulate at least 60 min of moderate-to-vigorous physical activity per day, sleep between 9 and 11 h per night, engage in no more than two hours of recreational screen time per day, spend several hours per day in light physical activity, while minimizing the time they spend sitting [69].

**Figure 4:**
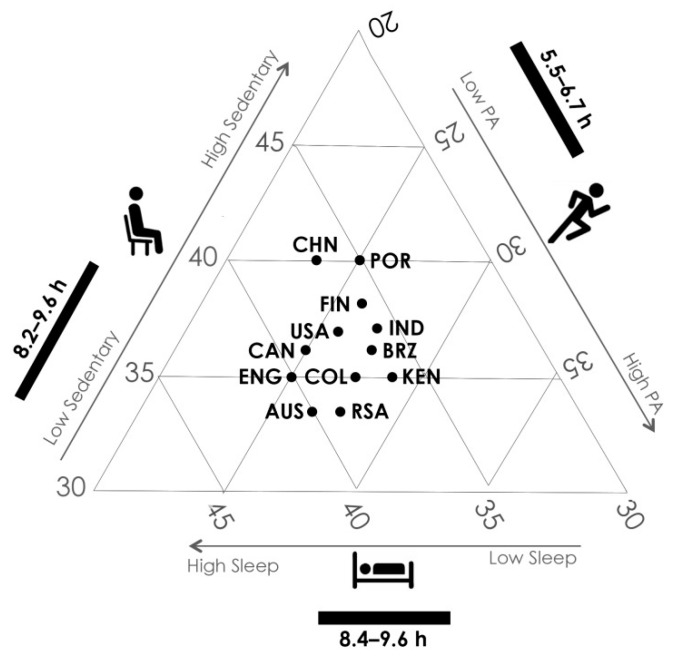
Ternary plot of the average proportions of the 24-h day spent in sleep (bottom axis), sedentary behaviour (left axis) and total physical activity (right axis) in the 12 ISCOLE countries. The black bars represent the range of time (h/day) spent in the various movement behaviours. For sedentary behavior, follow the direct horizontal line to the left axis; for physical activity, follow the lines sloping upwards from left to right to the right axis; for sleep, follow the lines sloping downwards from left to right to the bottom axis. Chinese (CHN) children, for example, spend on average 37% of the day sleeping, 40% of the day sedentary and 23% in physical activity. Compositional means are from Dumuid et al. [70]. AUS = Australia; BRZ = Brazil; CAN = Canada; CHN = China; COL = Colombia; ENG = England; FIN = Finland; IND = India; KEN = Kenya; POR = Portugal; RSA = Republic of South Africa; USA = United States.

Figure 4 presents estimates of the mean proportions (%) of the day spent in sleep, sedentary behavior, and total physical activity (light, moderate and vigorous) in children across the 12 ISCOLE sites [70]. The mean proportions of the day spent in the different behaviors is remarkably similar across countries given the expanded axes of the ternary plot, as one would predict from the proportion of the variance in these variables that is explained at the site level [42]. The mean time spent in the movement behaviors ranges from 508 min in Portugal to 579 in the United Kingdom for sleep, from 486 min in Australia to 573 min in China for sedentary time, and from 336 min in China to 406 min in Kenya for total physical activity [70]. In addition to these three variables that constitute a 24-h day, mean levels of moderate-to-vigorous physical activity ranges from 43 min in China to 69 min in Finland [70].

Associations between meeting combinations of the 24-h guidelines and obesity [71], health-related quality of life [72], and dietary patterns [73] have been explored in ISCOLE. Overall, the proportion of the sample meeting the overall recommendations (all three behaviors: moderate-to-vigorous physical activity, recreational screen time, and sleep duration) was 7%; individually, 44% of the sample met the physical activity recommendation, 39% met the screen time recommendation, and 42% met the sleep duration recommendation [71]. Meeting all three of the recommendations was associated with much lower odds of obesity (odds ratio = 0.28; 95% CI: 0.18–0.45) [71], higher health-related quality of life scores (51.2 vs. 50.0; *p* < 0.05) [72], higher healthy dietary pattern scores (0.18 vs. −0.01; *p* < 0.001), and lower unhealthy dietary pattern scores (−0.31 vs. −0.02; *p* < 0.001) [73].

A day is constrained by the 24-h period, which presents some challenges for the analysis of multiple movement behaviors together in relation to other health parameters such as obesity [74,75]. Traditional analyses that use Euclidian operations such as linear regression fail to account for the Aitchison geometry of the constrained space [75], and therefore compositional analysis approaches have been proposed as an alternative [76]. Noting the lack of suitable analytical techniques available to handle the analysis of 24-h movements behaviors, Dumuid et al. [77] developed a novel method for predicting change in a continuous outcome based on relative changes within a composition, and for calculating associated confidence intervals to allow for statistical inference. Using data from ISCOLE, we demonstrated the application of compositional multiple linear regression to estimate adiposity from children’s 24-h movement behaviors [77]. Furthermore, ISCOLE presented a unique opportunity to compare the results of traditional vs. compositional isotemporal substitution analyses in the associations between 24-h movement behaviors and obesity. The results of this investigation demonstrated that both compositional and traditional models estimated an unfavorable association with percentage body fat when time was reallocated from moderate-to-vigorous physical activity to any other behavior (sleep, sedentary behavior, light physical activity). However, unlike traditional models, compositional models found the differences in adiposity were (A) not necessarily symmetrical when an activity was being displaced, or displacing another movement behavior; (B) not linearly related to the durations of time reallocated; and (C) varied depending on the starting composition [75].

In an attempt to better understand the associations between lifestyle variables and obesity, Dumuid et al. [78] undertook a compositional cluster analysis in which the input variables including sedentary time, light, moderate and vigorous physical activity, sleep duration, TV viewing time, and healthy and unhealthy diet pattern scores were subjected to cluster analysis. Four clusters emerged and were labelled as: (A) Junk Food Screenies; (B) Actives; (C) Sitters; and (D) All-Rounders. Measures of adiposity varied across the clusters, and were highest in the Sitters and lowest in the Actives [78].

In addition to obesity, we have applied compositional data analysis to study the association between 24-h movement behaviors and health-related quality of life [70]. Relative to the other movement behaviors, the association was strongest with moderate-to-vigorous physical activity. Furthermore, this association was moderated by country-level HDI; the association between the moderate-to-vigorous physical activity and health-related quality of life was stronger among countries with a high HDI compared to countries with a lower HDI [70].

## 7. Other Novel Contributions of ISCOLE

In addition to the work described above, ISCOLE has made several other significant contributions to the literature.

### 7.1. Body Composition

The majority of the research conducted to date on associations between anthropometry and body fat in children has been in high-income countries, and there is a lack of data on associations in children from low- and middle-income countries. We explored the association between BMI and body fat (from bioelectric impedance) in ISCOLE [79]. Correlations between BMI and total body fat (kg) were >0.90 in all study sites, while correlations between BMI and percentage body fat (%) ranged from 0.76 to 0.96. Boys from India had higher percentage body fat than boys from several other countries at all levels of BMI, whereas Kenyan girls had lower levels of percentage body fat than girls from several other countries at all levels of BMI. Boys and girls from Colombia had higher values of percentage body fat at low levels of BMI, while Colombian boys at moderate and high levels of BMI also had higher values of percentage body fat than boys in other countries [79].

Given the difficulty in measuring height and weight in some field situations, we explored the utility of using mid-upper-arm circumference as an index of adiposity in ISCOLE [80]. Correlations between mid-upper-arm circumference and percentage body fat were 0.86 in girls (*p* < 0.001) and 0.88 in boys (*p* < 0.001) [80]. Furthermore, results from receiver operating characteristic (ROC) curves demonstrated areas under the curve (AUCs) for the prediction of obesity ≥0.97 in both boys and girls, suggesting that mid-upper-arm circumference may be a good screening tool for obesity and excess adiposity in resource-limited settings.

### 7.2. Identification of Physical Activity Thresholds

We used ROC analyses to estimate the optimal thresholds of moderate-to-vigorous physical activity that were related to the identification of obesity in ISCOLE [43]. The results indicated that the optimal thresholds were 55 (95% CI: 50–64) minutes per day in the total sample, 65 (95% CI: 55–75) minutes per day in boys, and 49 (95% CI: 43–62) minutes per day in girls [43]. These thresholds are comparable to the global physical activity recommendation, which call for children to accumulate at least 60 min per day of moderate-to-vigorous physical activity [51].

Given the recent interest in the health effects associated with sedentary behavior, and the possible interaction between sedentary behavior and physical activity on health outcomes [81], we attempted to identify the optimal thresholds of moderate-to-vigorous physical activity at different levels of sedentary behavior [82]. The results showed that the optimal thresholds of moderate-to-vigorous physical activity to predict obesity ranged from 37.9 to 75.9 min per day in boys and from 32.5 to 62.7 min per day in girls across levels of sedentary behavior [82]. The incorporation of sedentary behavior did not alter or improve the prediction of obesity in this sample, suggesting that the current physical activity guidelines may apply broadly to all children, regardless of their level of sedentary behavior.

### 7.3. Inequality in Lifestyle and Obesity

Most studies examining associations between obesity and movement behaviors such as physical activity, sedentary behavior, and sleep duration have focused on average values, despite important within and between country variability in these behaviors. Using data from the accelerometers in smartphones in a sample of adults distributed across 111 countries, Althoff et al. [83] found that country-level inequality in physical activity (quantified using the Gini coefficient applied to the accelerometer steps/day data) was a better correlate of obesity prevalence than average physical activity volume (mean steps/day). We explored this issue in ISCOLE, and expanded the focus to include sedentary behavior and sleep as potential correlates of obesity [84].

**Figure 5:**
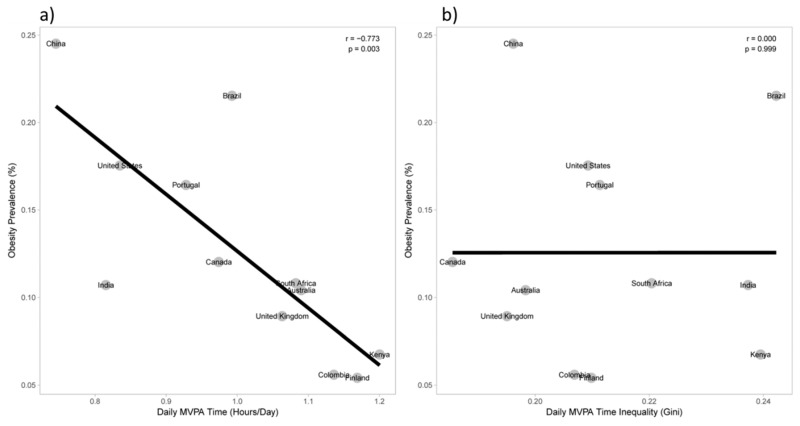
Association between moderate-to-vigorous physical activity (MVPA) and obesity. (**a**) shows the correlation between average MVPA and obesity, while (**b**) shows the correlation between MVPA inequality (Gini coefficient) and obesity. Boys and girls are combined for analysis. Correlation coefficients were compared using a Steiger’s Z-test (*p* = 0.029), adapted from Chaput et al. [84].

Our results showed that average moderate-to-vigorous physical activity (hours/day) was a better correlate of obesity than moderate-to-vigorous physical activity inequality (r = −0.77 vs. r = 0.00, *p* = 0.03) [84] (see Figure 5). Along the same lines, average sedentary time (hours/day) was also a better correlate of obesity than sedentary time inequality (r = 0.52 vs. r = 0.32, *p* = 0.05). The differences in associations for mean vs. inequality measures for screen time and sleep period time were not statistically significant [84]. Although there is promise in further exploring associations between inequality in lifestyle behaviors and health outcomes, our results suggest that mean estimates of behavior are still important correlates of obesity in children.

## 8. Summary of Research Contributions

As described above, ISCOLE has made many significant research contributions related to understanding global patterns of obesity across countries at different levels of human development and identifying correlates of obesity, physical activity, sedentary behavior, and dietary intake. Furthermore, ISCOLE generated several important methodological advances to the field over the course of the study. Table 2 provides a summary of the major research contributions made by ISCOLE related to the global childhood obesity epidemic. The results from ISCOLE can help inform the development of interventions targeting promising correlates of obesity in different settings. Some findings were robust across all study sites; while other findings were limited to either higher or lower income countries. A careful examination of the patterns of results across countries will be required to deploy the most effective intervention in a given setting.

## 9. A Platform for International Training, Data Entry, and Data Quality for Multi-Country Research Studies

In addition to its scientific contributions, ISCOLE provided a platform for research capacity development around the world. The study was governed by a standardized protocol implemented using strict quality control procedures, including in-person and online study personnel training, site visits, and remote source data verification. All investigators shared responsibility for quality control. A major component of study management was a shared website, which allowed access to all study documents, training materials, remote data entry, accelerometer data uploads, and real-time data validation. This system, based on a common data model, facilitated timely communication and data transfers between the sites and coordinating center. The enrollment target for ISCOLE was 6000 children; however, the final sample size was 7372. We believe the successful recruitment and high data quality are the result of well trained and prepared research staff and co-ownership and investigator investment in the study. Over 240 people have worked on ISCOLE, including senior and junior faculty, post-doctoral fellows, students, and research staff. To date, 25 students (12 doctoral, 10 masters and 3 undergraduate) from 10 countries have used ISCOLE as the foundation for their thesis. ISCOLE has had a tremendous impact on developing research capacity in countries spanning a wide range of HDI.

## 10. Strengths and Limitations

There are several strengths and limitations associated with the design and implementation of ISCOLE. Marked strengths of the study include the implementation of a standardized research protocol using the same instruments (adapted to the local context as required) and equipment at all study sites, and the inclusion of research sites and investigators from countries that varied widely in human development [7]. Furthermore, the deployment of a web-based data collection and staff training infrastructure that allowed for real-time data entry and verification increased the assurance of quality data. The publication of the study questionnaires, protocols and data algorithms in peer-reviewed journals, which allows for transparency and reproducibility of the results, is another strength of the study. Such resources are also immensely beneficial to other researchers interested in similar or related research. The timely and thorough publication and presentation of ISCOLE findings helped maximize the dissemination and impact of the research, including, but not limited to the notable scientific and methodological advances discussed in this paper. ISCOLE also contributed markedly to capacity building via the personal skill development of research trainees.

There are several limitations of ISCOLE that warrant discussion. First, the fundamental design of ISCOLE is a cross-sectional study, which limits inferences about cause-and-effect relationships. Second, the ISCOLE study sites represent urban and semi-urban populations, and the samples do not include children from rural areas. The decision to exclude rural samples was based on logistical limitations related to data collection; further research is required to better understand urban-rural differences in the correlates of obesity and related behaviors in multi-national studies. This is of particular importance in low- and middle-income countries where the majority of people live in rural environments. We deployed a rigorous research protocol; however, the assessment of dietary intake in free-living children remains a challenge. We used a validated and widely used FFQ to assess dietary intake patterns; however, the FFQ was short and we were unable to precisely quantify dietary intake (kcals, macronutrients, etc.). Furthermore, research is required to develop better methods of dietary assessment in children.

## 11. A Transparent Model for Public-Private Research Partnerships

ISCOLE was funded by the Coca-Cola Company through a research contract with PBRC. PBRC, in turn, executed sub-contracts with each of the study site institutions. With the exception of requiring that the study be global in nature, the funder had no role in the design and conduct of the study; collection, management, analysis and interpretation of the data, preparation of manuscripts, and the decision whether to publish the results or not. The overall study design, protocol, and all study procedures were developed solely by the principal investigators, co-investigators and research staff. During the period of the research contract (2011–2014), the research team provided regular updates to the funder about the study progress on a quarterly basis. These reports focused on the achievement of operational milestones such as completion of protocols, staff training, field site implementation, achieving recruitment targets, remote site monitoring, and progress on data management.

Given the increased scrutiny associated with industry-sponsored research, we took several precautions to ensure the integrity of the study methods and results. First, we established an External Advisory Board that was charged with assessing the overall progress, rigor and objectivity of ISCOLE, as well as providing an unbiased assessment of the science and the role of the sponsor. Second, we published the design and methods of ISCOLE in an open-access journal, and included the questionnaires and survey instruments in an online appendix [7]. Third, we have made our accelerometer manual of procedures and algorithms freely available online so that they can be tested, replicated and utilized by other scientists [14,16,17]. Fourth, wherever possible, we have published the results from ISCOLE in open-access, peer-reviewed journals so that they can be freely available to a wide audience. Furthermore, all scientific presentations and publications have clearly acknowledged the funding source and the role of the funder. To date, we have published more than 100 scientific papers that have all undergone peer-review and have been found worthy to make significant contributions to the extant literature. Moreover, the large number of students and young researchers who were engaged in ISCOLE highlights the research capacity building that occurred across low-, middle- and high-income countries. Finally, we have declared our data available upon reasonable request for researchers who wish to replicate or challenge our research findings.

Despite our concerted efforts to ensure transparency and rigor in the design and conduct of ISCOLE, we experienced skepticism about the role of the sponsor. We have received several Freedom of Information requests for access to emails from the study investigators and the sponsor. To date, we have provided emails to several media organizations and the US Right to Know organization. This correspondence was the subject of a manuscript that attempted to understand the relationship between “an industry sponsor and public health academics” [85]. The primary conclusion of the study in the first line of the conclusions section is that “Overall, apart from influencing the total number of study sites, we found no evidence of Coca-Cola exerting ‘hard power’ over the Pennington PIs, where the funder directly changes core methodological principles or points in the research” [85]. The authors tried to make the case that the funder was involved in the study design since they negotiated the number of study sites with the principal investigators as we developed the budget. However, we maintain that the overall study design was not impacted by the number of study sites, i.e., whether the study included 12, 13 or 14 countries; rather, this was purely a budgetary issue, where the budget largely drove the number of study sites that could be recruited [86]. The selection of study sites was at the discretion of the principal investigators. While the funder made suggestions about potential sites to include in order to ensure global representation, the final slate of study sites was selected solely by the principal investigators, and did not include specific sites recommended by the funder.

In summary, the ISCOLE investigators acted in good faith while developing and executing the ISCOLE protocol. Despite a high level of scrutiny (and review of thousands of our emails) from the media and other organizations, we have conducted ISCOLE with transparency and integrity and we are immensely proud of it. We hope that such scrutiny of our work will not dissuade other researchers from developing appropriately managed and transparent public-private partnerships to tackle important public health issues. We feel that ISCOLE represents a successful and transparent model for future public-private partnerships with academics.

## 12. Conclusions

ISCOLE was a collaboration of scientists, students and staff from 12 countries ranging widely in levels of human and economic development. Using a model of shared ownership, ISCOLE surpassed all recruitment and quality control goals. To date, more than 100 peer-reviewed papers have been published from ISCOLE. In addition to being an engine of research capacity development, ISCOLE has made many significant contributions to our understanding of the global childhood obesity epidemic in a short period of time. The findings of ISCOLE could, in turn, inform global efforts, such as the World Health Organization Global Action Plan on Physical Activity 2018–2030 [87], and the achievement of the United Nation’s Sustainable Development Goals [88]. Furthermore, the results from ISCOLE can inform the development of culturally tailored interventions that can be deployed and tested across a range of settings, and we encourage future collaborations that will build upon ISCOLE to improve the health of children across the world.

## Figures and Tables

**Table 1:** Descriptive characteristics of the study sample from the International Study of Childhood Obesity, Lifestyle and the Environment (ISCOLE).

Study Site	HDI *	Boys (*n*)	Girls (*n*)	Age (year) **	NW (%)	OV (%)	OB (%)	Parent Education (%)		
								1	2	3
**China (Tianjin)**	**0.687**	**293**	**259**	**9.9 (0.4)**	**58.9**	**17.4**	23.7	33.0	44.4	22.6
Brazil (Sao Paulo)	0.718	287	297	10.5 (0.5)	56.3	22.8	20.9	24.3	52.8	22.9
United States (Baton Rouge)	0.910	281	370	10.0 (0.6)	58.8	22.4	18.7	8.9	44.6	46.6
Portugal (Porto)	0.809	358	419	10.4 (0.3)	52.8	29.7	17.5	46.7	32.8	20.5
Canada (Ottawa)	0.908	239	328	10.5 (0.4)	69.3	18.9	11.8	2.0	27.7	70.4
South Africa (Cape Town)	0.619	223	327	10.3 (0.7)	73.6	15.6	10.7	48.0	39.0	12.9
Australia (Adelaide)	0.929	243	285	10.7 (0.4)	62.1	27.5	10.4	11.4	47.7	40.9
India (Bangalore)	0.547	292	328	10.4 (0.5)	66.3	23.4	10.3	4.8	21.7	73.4
United Kingdom (Bath)	0.863	237	288	10.9 (0.5)	69.7	20.6	9.7	3.0	51.6	45.4
Kenya (Nairobi)	0.509	262	301	10.2 (0.7)	78.9	14.6	6.6	13.9	45.7	40.4
Colombia (Bogota)	0.710	454	465	10.5 (0.6)	77.2	17.1	5.8	31.8	50.7	17.5
Finland (Helsinki)	0.882	253	283	10.5 (0.4)	76.3	18.3	5.4	2.8	55.1	42.1

* Human Development Index [10]; ** Mean (SD); NW: normal weight; OV: overweight; OB: obese. Parent education levels are 1 <high school and some high school, 2 completed high school and some post-secondary (e.g., vocational diploma or certificate); 3 bachelor degree and post-graduate.

**Table 2:** Major research contributions of the International Study of Childhood Obesity, Lifestyle and the Environment (ISCOLE) to understanding the global obesity epidemic.

Research Area	Major Contribution
Global Patterns of Obesity and Related Behaviors	There is evidence for global epidemiological transitions in obesity and physical activity across countries at different levels of human development; however, there is less evidence for epidemiological transitions in dietary behaviors and sleep duration Inequality in lifestyle behaviors is not a better correlate of obesity than mean levels of lifestyle behaviors in countries at different levels of human development
Correlates of Obesity	The proportion of the variance in BMI and waist circumference explained at the individual level is greater than 90%, with the remainder being explained at the school and site levels Moderate-to-vigorous physical activity is a robust correlate of obesity across all study sites; active school transportation was also related to a lower odds of obesity Sedentary behavior and TV viewing are both related to a higher odds of obesity General dietary patterns (healthy/unhealthy) are not related to obesity; however, regular breakfast consumption was related to a lower odds of obesity; regular soft drink consumption was related to a higher odds of obesity in boys and diet soft drink consumption was related to a higher odds of obesity in girls Parental overweight, gestational diabetes and high birth weight are related to a higher odds of obesity; high moderate-to-vigorous activity and low sedentary time seem to negate the effects of birthweight on childhood obesity Meeting all three 24-h movement guidelines (moderate-to-vigorous physical activity, TV viewing, sleep) was associated with much lower odds of obesity
Correlates of Physical Activity & Sedentary Behavior	Participation in physical education classes is associated with higher levels of moderate-to-vigorous physical activity and less sedentary behavior Active transportation to school is associated with higher weekday moderate-to-vigorous physical activity and lower light physical activity before school There is wide variability in the correlates of active school transport across study sites. Longer trip duration was associated with lower odds of active school transportation in eight sites; whereas individual and neighborhood factors were associated with active school transportation in three sites or less Children with at least one piece of electronic media in their bedroom had lower levels of moderate-to-vigorous physical activity More frequent physical activity in the home and yard, ownership of more frequently used play equipment, and higher social support for physical activity were associated with higher moderate-to-vigorous physical activity; the association between play equipment ownership and moderate-to-vigorous physical activity varied across sites Collective efficacy was inversely associated with moderate-to-vigorous physical activity among children in low/lower-middle-income countries, while it was positively associated with moderate-to-vigorous physical activity among children in high-income countries. Perceived crime was significantly associated with lower moderate-to-vigorous physical activity in high-income countries but not in low/lower-middle-income countries or upper-middle-income countries Common correlates of sedentary time and TV viewing time were excess weight status, not meeting physical activity recommendations, and having a TV in the bedroom Greater time spent outdoors was associated with higher moderate-to-vigorous and light physical activity, and lower sedentary time
Correlates of Dietary Intake	More meals eaten outside home and school was associated with higher unhealthy diet pattern scores Low availability of empty-calorie foods at home was more important than high availability of wholesome foods for a lower unhealthy diet pattern Availability of wholesome foods at home was positively associated with a healthy diet pattern; food availability at school was not associated with the dietary patterns Sleep duration and sleep efficiency were negatively associated with an unhealthy diet Shorter sleep duration was associated with higher intake of regular soft drinks, while earlier bedtimes were associated with lower intake of regular soft drinks and higher intake of energy drinks and sports drinks Meeting all three 24-h movement guidelines (moderate-to-vigorous physical activity, TV viewing, sleep) was associated with higher healthy dietary pattern scores and lower unhealthy dietary pattern scores
Methodological Advances	Creation and validation of an automated algorithm to determine sleep parameters from 24-h waist-worn accelerometry Development and application of a novel compositional data analysis approach to be used with 24-h movement behavior data Adaptation and reliability assessment of a school environmental audit tool Adaptation and validation of a food frequency questionnaire for use in different cultural settings

## Supplementary Materials

The following are available online at https://www.mdpi.com/2072-6643/11/4/848/s1, File S1: ISCOLE Publications List.
