# Supplementary material for: International Study of Childhood Obesity, Lifestyle and the Environment (ISCOLE): Contributions to Understanding the Global Obesity Epidemic
Source: Nutrients. 2019 Apr 15;11(4):848. doi: 10.3390/nu11040848 (PMC6521223; doi:10.3390/nu11040848)
Supplement: Supplementary file 1 [file nutrients-11-00848-s001.pdf]

Supplementary Material:

File S1: Iscole Publications

2013

1. Katzmarzyk P.T., T.V. Barreira, S.T. Broyles, C.M. Champagne, J.-P. Chaput, M. Fogelholm, G. Hu, W.D. Johnson, R. Kuriyan, A. Kurpad, E.V. Lambert, J. Maia, V. Matsudo, C. Maher, T. Olds, V. Onywera, O.L. Sarmiento, M. Standage, M.S. Tremblay, C. Tudor-Locke, P. Zhao and T.S. Church. The International Study of Childhood Obesity, Lifestyle and the Environment (ISCOLE): Design and Methods. *BMC Public Health* 2013;13:900.

2014

2. Borghese M., M.S. Tremblay, G. Leduc, C. Boyer, P. Belanger, A.G. LeBlanc, C. Francis and J.-P. Chaput. Independent and combined associations of total sedentary time and television viewing time with food intake patterns of 9-11 year-old Canadian children. *Applied Physiology, Nutrition and Metabolism* 2014;39:937-943.
3. Chaput J.-P., G. Leduc, C. Boyer, P. Belanger, A.G. LeBlanc, M.M. Borghese, and M.S. Tremblay. Electronic screens in children's bedrooms and adiposity, physical activity and sleep: Do the number and type of electronic devices matter? *Canadian Journal of Public Health* 2014;105(4):e273-e279.
4. Chaput J.-P., G. Leduc, C. Boyer, P. Belanger, A.G. LeBlanc, M.M. Borghese and M.S. Tremblay. Objectively measured physical activity, sedentary time and sleep duration: independent and combined associations with adiposity in Canadian children. *Nutrition and Diabetes* 2014;4:1-5.
5. Gomes T.N., F.K. dos Santos, D. Santos, S. Pereira, R.N. Chaves, P.T. Katzmarzyk and J.A.R. Maia. Correlates of sedentary behaviour in children. A multilevel modelling approach. *BMC Public Health* 2014;14:890.
6. Gomes T.N., F.K. dos Santos, M. Souza, S. Pereira, P.T. Katzmarzyk and J.A.R. Maia. Overweight and obesity in Portuguese children: Prevalence and correlates. *International Journal of Environmental Research and Public Health* 2014;11:11398-11417.
7. Larouche R., J.P. Chaput, G. Leduc, C. Boyer, P. Belanger, A.G. Leblanc, M.M. Borghese and M.S. Tremblay . A cross-sectional examination of socio-demographic and school-level correlates of children's school travel mode in Ottawa, Canada. *BMC Public Health* 2014;14:497.
8. Muthuri S.K., L-J M. Wachira, V.O. Onywera and M.S. Tremblay. Comparative study of physical activity patterns among school children in Kenya and Canada: Results from the ISCOLE project. *African Journal for Physical Health Education, Recreation and Dance* 2014;20(2:2):765-779.
9. Muthuri S.K., J.L. Wachira, V.O. Onywera and M.S. Tremblay. Correlates of objectively measured overweight/obesity and physical activity in Kenyan school children: Results from ISCOLE-Kenya. *BMC Public Health* 2014;14:436.
10. Tudor-Locke C., T.V. Barreira, J.M. Schuna, E.F. Mire and P.T. Katzmarzyk. Fully automated waist-worn accelerometer algorithm for detecting children's sleep period time separate from 24-h physical activity or sedentary behaviors. *Applied Physiology, Nutrition and Metabolism* 2014; 39:53-57.

2015

11. Barreira T.V., J.M. Schuna, E.F. Mire, P.T. Katzmarzyk J-P Chaput, G. Leduc and C. Tudor-Locke. Identifying children's nocturnal sleep using 24-hour waist accelerometry. *Medicine & Science in Sports & Exercise* 2015;47:937-943.
12. Barreira T.V., J.M. Schuna, C. Tudor-Locke, J.-P. Chaput, T.S. Church, M. Fogelholm, G. Hu, R. Kuriyan, A. Kurpad, E.V. Lambert, C. Maher, J. Maia, V. Matsudo, T. Olds, V. Onywera, O.L. Sarmiento, M. Standage, M.S. Tremblay, P. Zhao and P.T. Katzmarzyk for the ISCOLE Research Group. Reliability of accelerometer-determined physical activity and sedentary behavior in school-aged children: A 12 country study. *International Journal of Obesity Supplements* 2015;5(2):S29-S35.
13. Borges A., T.N. Gomes, D.V. Santos, S. Pereira, F.K. Santos, R.N. Chaves, P.T. Katzmarzyk and J.A.R. Maia. A count model to study correlates of 60 minutes of daily physical activity in Portuguese children. *International Journal of Environmental Research and Public Health* 2015;12:2557-2573.
14. Borghese M.M., M.S. Tremblay, P.T. Katzmarzyk, C. Tudor-Locke, J.M. Schuna, G. Leduc, C. Boyer, A.G. LeBlanc, and J.-P. Chaput. Mediating role of television time, diet patterns, physical activity and sleep duration in the association between television in the bedroom and adiposity in 10-year-old children. *International Journal of Behavioral Nutrition and Physical Activity* 2015;12:60 doi:10.1186/s12966-015-0221-5.
15. Borghese M.M., M.S. Tremblay, G. Leduc, C. Boyer, P. Bélanger, A.G. LeBlanc, C. Francis and J.-P. Chaput. Television viewing and food intake during television viewing in normal weight, overweight, and obese 9-11 year-old Canadian Children: A cross-sectional analysis. *Journal of Nutritional Science* 2015;4:e8:1-9.
16. Broyles S.T., K.D. Denstel, T.S. Church, J.-P. Chaput, M. Fogelholm, G. Hu, R. Kuriyan, A. Kurpad, E.V. Lambert, C. Maher, J. Maia, V. Matsudo, T. Olds, V. Onywera, O.L. Sarmiento, M. Standage, M.S. Tremblay, C. Tudor-Locke, P. Zhao and P.T. Katzmarzyk for the ISCOLE Research Group. The epidemiological transition and the global childhood obesity epidemic. *International Journal of Obesity Supplements* 2015;5(2):S3-S8.
17. Broyles S.T., K.T. Drazba, T.S. Church, J.-P. Chaput, M. Fogelholm, G. Hu, R. Kuriyan, A. Kurpad, E.V. Lambert, C. Maher, J. Maia, V. Matsudo, T. Olds, V. Onywera, O.L. Sarmiento, M. Standage, M.S. Tremblay, C. Tudor-Locke, P. Zhao and P.T. Katzmarzyk for the ISCOLE Research Group. Development and reliability of an audit tool to assess the school physical activity environment across 12 countries. *International Journal of Obesity Supplements* 2015;5(2):S36-S42.
18. Chaput J.P., P.T. Katzmarzyk, A.G. LeBlanc, M.S. Tremblay, T.V. Barreira, S.T. Broyles, M. Fogelholm, G. Hu, R. Kuriyan, A. Kurpad, E.V. Lambert, D.E. Rae, C. Maher, J. Maia, V. Matsudo, V. Onywera, O.L. Sarmiento, M. Standage, C. Tudor-Locke, P. Zhao, and T. Olds for the ISCOLE Research Group. Associations between sleep patterns and lifestyle behaviors in children: An international comparison. *International Journal of Obesity Supplements* 2015;5(2):S59-S65.
19. Denstel K.D., S.T. Broyles, R. Larouche, O.L. Sarmiento, T.V. Barreira, J.-P. Chaput, T.S. Church, M. Fogelholm, G. Hu, R. Kuriyan, A. Kurpad, E.V. Lambert, C. Maher, J. Maia, V. Matsudo, T. Olds, V. Onywera, M. Standage, M.S. Tremblay, C. Tudor-Locke, P. Zhao and P.T. Katzmarzyk for the ISCOLE Research Group. Active school transport and weekday physical activity in 9-11 year old children from 12 countries. *International Journal of Obesity Supplements* 2015;5(2):S100-S106.

20. Ferrari G., T.L. Araujo, L.C. Oliveira, V. Matsudo, T.V. Barreira, E. Mire, C. Tudor-Locke and P.T. Katzmarzyk. Association between television viewing and physical activity in 10-year old Brazilian children. *Journal of Physical Activity and Health* 2015: 12:1401-1408.
21. Ferrari G., T.L. Araujo, L.C. Oliveira, V. Matsudo and M. Fisberg. Association between electronic equipment in the bedroom and sedentary lifestyle, physical activity and body mass index of children. *Jornal de Pediatria* 2015;91(6):574-582.
22. Ferrari G., L.C. Oliveira, T.L. Araujo, V. Matsudo, T.V. Barreira, C. Tudor-Locke and P.T. Katzmarzyk. Moderate-to-vigorous physical activity and sedentary behavior: independent associations with body composition variables in Brazilian children. *Pediatric Exercise Science* 2015;27:380-389.
23. Gomes T.N., D. Hedeker, F.K. dos Santos, S. Pereira, P.T. Katzmarzyk and J.A.R. Maia. Why are children different in their daily sedentariness? An approach based on the mixed-effects location scale model. *PloS One* 2015;10(7):e0132192.
24. Gomes T.N., P.T. Katzmarzyk, F.K. dos Santos, R. Chaves, D.M. Santos, S. Pereira, C.M. Champagne, D. Hedeker and J.A.R. Maia. Are BMI and sedentariness correlated? A multilevel study in children. *Nutrients* 2015;7:5889-5904.
25. Katzmarzyk P.T., T.V. Barreira, S.T. Broyles, C.M. Champagne, J.-P. Chaput, M. Fogelholm, G. Hu, W.D. Johnson, R. Kuriyan, A. Kurpad, E.V. Lambert, C. Maher, J. Maia, V. Matsudo, T. Olds, V. Onywera, O.L. Sarmiento, M. Standage, M.S. Tremblay, C. Tudor-Locke, P. Zhao and T.S. Church for the ISCOLE Research Group. Relationship between lifestyle behaviors and obesity in children ages 9-11. Results from a 12-country study. *Obesity* 2015;23:1696-1702.
26. Katzmarzyk P.T., T.V. Barreira, S.T. Broyles, C.M. Champagne, J.-P. Chaput, M. Fogelholm, G. Hu, W.D. Johnson, R. Kuriyan, A. Kurpad, E.V. Lambert, C. Maher, J. Maia, V. Matsudo, T.S. Olds, V. Onywera, O.L. Sarmiento, M. Standage, M.S. Tremblay, C. Tudor-Locke, P. Zhao and T.S. Church for the ISCOLE Research Group. Physical activity, sedentary time and obesity in an international sample of children. *Medicine and Science in Sports and Exercise* 2015;47:2062-2069.
27. Katzmarzyk P.T., T.V. Barreira, S.T. Broyles, J.-P. Chaput, M. Fogelholm, G. Hu, R. Kuriyan, A. Kurpad, E.V. Lambert, C. Maher, J. Maia, V. Matsudo, T. Olds, V. Onywera, O.L. Sarmiento, M. Standage, M.S. Tremblay, C. Tudor-Locke, P. Zhao and T.S. Church for the ISCOLE Research Group. Association between body mass index and body fat in 9-11 year old children from countries spanning a range of human development. *International Journal of Obesity Supplements* 2015;5(2):S43-S46.
28. Larouche R., O.L. Sarmiento, S.T. Broyles, K.D. Denstel, T.S. Church, J.-P. Chaput, M. Fogelholm, G. Hu, R. Kuriyan, A. Kurpad, E.V. Lambert, C. Maher, J. Maia, V. Matsudo, T. Olds, V. Onywera, M. Standage, M.S. Tremblay, C. Tudor-Locke, P. Zhao and P.T. Katzmarzyk for the ISCOLE Research Group. Are the correlates of active school transport context-specific? *International Journal of Obesity Supplements* 2015;5(2):S89-S99.
29. LeBlanc A.G., S.T. Broyles, Harrington D., J.P. Chaput, M. Fogelholm, G. Hu, R. Kuriyan, A. Kurpad, E.V. Lambert, C. Maher, J. Maia, V. Matsudo, T. Olds, V.O. Onywera, O.L. Sarmiento, M. Standage, M.S. Tremblay, C. Tudor-Locke, P. Zhao, T.S. Church and P.T. Katzmarzyk. Correlates of total sedentary time and screen time in 9-11 year-old children around the world: The International Study of Childhood Obesity, Lifestyle, and the Environment. *PLoS One* 2015;10(6):e0129622.

30. LeBlanc A.G., S.T. Broyles, J.-P. Chaput, G. Leduc, C. Boyer, M.M. Borghese and M.S. Tremblay. Correlates of objectively measured sedentary time and self-reported screen time in Canadian children. *International Journal of Behavioral Nutrition and Physical Activity* 2015;12:38 doi:10.1186/s12966-015-0197-1.
31. LeBlanc A.G., P.T. Katzmarzyk, T.V. Barreira, S.T. Broyles, J.-P. Chaput, T.S. Church, M. Fogelholm, D.M. Harrington, G. Hu, R. Kuriyan, A. Kurpad, E.V. Lambert, C. Maher, J. Maia, V. Matsudo, T. Olds, V. Onywera, O.L. Sarmiento, M. Standage, C. Tudor-Locke, P. Zhao and M.S. Tremblay for the ISCOLE Research Group. Are participant characteristics from ISCOLE study sites comparable to the rest of their country? *International Journal of Obesity Supplements* 2015;5(2):S9-S16.
32. McNeil J., M.S. Tremblay, G. Leduc, C. Boyer, P. Belanger, A.G. LeBlanc, M.M. Borghese and J.-P. Chaput. Objectively-measured sleep and its association with adiposity and physical activity in a sample of Canadian children. *Journal of Sleep Research* 2015;24:131-139.
33. Mikkilä V., H. Vepsäläinen, T. Saloheimo, S.A. Gonzalez, J.D. Meisel, G. Hu, C.M. Champagne, T.S. Church, P.T. Katzmarzyk, R. Kuriyan, A. Kurpad, E.V. Lambert, C. Maher, J. Maia, V. Matsudo, T.S. Olds, V. Onywera, O.L. Sarmiento, M. Standage, M.S. Tremblay, C. Tudor-Locke, P. Zhao and M. Fogelholm for the ISCOLE Research Group. An international comparison of dietary patterns in 9-11 year old children. *International Journal of Obesity Supplements* 2015;5(2):S17-S21.
34. Muthuri S.K., J.L M. Wachira, V.O. Onywera and M.S. Tremblay. Direct and self-reported measures of physical activity and sedentary behaviours by weight status in school-aged children: Results from ISCOLE-Kenya. *Annals of Human Biology 2015;*42(3):237-245.
35. Pereira S., T.N. Gomes, A. Borges, D.V. Santos, M. Souza, F.K. dos Santos, R.N. Chaves, P.T. Katzmarzyk and J.A.R. Maia. Variability and stability in daily moderate-to-vigorous physical activity among 10 year old children. *International Journal of Environmental Research and Public Health* 2015;12:9248-9263.
36. Pereira S., P.T. Katzmarzyk, T.N. Gomes, A. Borges, D.V. Santos, M. Souza, F.K. dos Santos, R.N. Chaves, T.V. Barreira and J.A.R. Maia. Profiling physical activity, diet, screen and sleep habits in Portuguese children. *Nutrients* 2015;7:4345-4362.
37. Qiao Y., J. Ma, Y. Wang, W. Li, P.T. Katzmarzyk, J.-P. Chaput, M. Fogelholm, W.D. Johnson, R. Kuriyan, A. Kurpad, E.V. Lambert, C. Maher, J. Maia, V. Matsudo, T. Olds, V. Onywera, O.L. Sarmiento, M. Standage, M.S. Tremblay, C. Tudor-Locke, T.S. Church, P. Zhao and G. Hu for the ISCOLE Research Group. Birth weight and childhood obesity: A 12-country study. *International Journal of Obesity Supplements* 2015;5(2):S74-S79.
38. Saloheimo T., S.A. González, M. Erkkola, D.M. Milauskas, J.D. Meisel, C.M. Champagne, C. Tudor-Locke, O.L. Sarmiento, P.T. Katzmarzyk and M. Fogelholm for the ISCOLE Research Group. The reliability and validity of a short food frequency questionnaire among 9-11 year olds: A multinational study on 3 middle income and high income countries. *International Journal of Obesity Supplements* 2015;5(2):S22-S28.
39. Sarmiento O.L., P. Lemoine, S.A. Gonzalez, S.T. Broyles, K.D. Denstel, R. Larouche, V. Onywera, T.V. Barreira, J.-P. Chaput, M. Fogelholm, G. Hu, R. Kuriyan, A. Kurpad, E.V. Lambert, C. Maher, J. Maia, V. Matsudo, T. Olds, M. Standage, M.S. Tremblay, C. Tudor-Locke, P. Zhao, T.S. Church and P.T. Katzmarzyk for the ISCOLE Research Group. Relationships between active school transport and

adiposity indicators in school age children from low-, middle- and high-income countries. *International Journal of Obesity Supplements* 2015;5(2):S107-S114.

40. Tudor-Locke C., T.V. Barreira, J.M. Schuna, Jr. and P.T. Katzmarzyk for the ISCOLE Research Group. Unique contributions of ISCOLE to the advancement of accelerometry in large studies. *International Journal of Obesity Supplements* 2015;5(2):S53-S58.

41. Tudor-Locke C., T.V. Barreira, J.M. Schuna, E.F. Mire, J.P Chaput, M. Fogelholm, G. Hu, R. Kuriyan, A. Kurpad, E.V. Lambert, C. Maher, J. Maia, V. Matsudo, T. Olds, V. Onywera, O.L. Sarmiento, M. Standage, M.S. Tremblay, P. Zhao, T.S. Church and P.T. Katzmarzyk. Improving wear-time compliance with a 24-hour waist-worn accelerometry protocol in the International Study of Childhood Obesity, Lifestyle, and the Environment (ISCOLE). *International Journal of Behavioral Nutrition and Physical Activity* 2015; 12 (1) 172.

42. Tudor-Locke C., E.F. Mire, T.V. Barreira, J.M. Schuna Jr., J.-P. Chaput, M. Fogelholm, G. Hu, A. Kurpad, R. Kuriyan, E.V. Lambert, C. Maher, J. Maia, V. Matsudo, T. Olds, V. Onywera, O.L. Sarmiento, M. Standage, M.S. Tremblay, P. Zhao, T.S. Church and P.T. Katzmarzyk for the ISCOLE Research Group. Nocturnal sleep-related variables from 24-hour free-living waist-worn accelerometry: International Study of Childhood, Obesity, Lifestyle and the Environment (ISCOLE). *International Journal of Obesity Supplements* 2015;5(2):S47-S52.

43. Tudor-Locke C., E.F. Mire, K.N. Dentro, T.V. Barreira, J.M. Schuna, P. Zhao, M.S. Tremblay, M. Standage, O.L. Sarmiento, V. Onywera, T. Olds, V. Matsudo, J. Maia, C. Maher, E.V. Lambert, A. Kurpad, R. Kuriyan, G. Hu, M. Fogelholm, J.P. Chaput, T.S. Church and P.T. Katzmarzyk. A model for presenting accelerometer paradata in large studies: ISCOLE. *International Journal of Behavioral Nutrition and Physical Activity* 2015; 12:52.

44. Vepsäläinen H., V. Mikkilä, M. Erkkola, S.T. Broyles, J.-P. Chaput, G. Hu, R. Kuriyan, A. Kurpad, E.V. Lambert, C. Maher, J. Maia, V. Matsudo, T. Olds, V. Onywera, O.L. Sarmiento, M. Standage, M.S. Tremblay, C. Tudor-Locke, P. Zhao, T.S. Church, P.T. Katzmarzyk and M. Fogelholm for the ISCOLE Research Group. Association between home and school food environments and dietary patterns among 9-11 year old children in 12 countries. *International Journal of Obesity Supplements* 2015;5(2):S66-S73.

45. Zakrzewski J.K., F.B. Gillison, S. Cumming, T.S. Church, P.T. Katzmarzyk, S.T. Broyles, C.M. Champagne, J.-P. Chaput, K. D. Denstel, M. Fogelholm, G. Hu, R. Kuriyan, A. Kurpad, E.V. Lambert, C. Maher, J. Maia, V. Matsudo, E.F. Mire, T. Olds, V. Onywera, O.L. Sarmiento, M.S. Tremblay, C. Tudor-Locke, P. Zhao and M. Standage for the ISCOLE Research Group. Associations between breakfast frequency and adiposity indicators in children from 12 countries. *International Journal of Obesity Supplements* 2015;5(2):S80-S88.

<u>2016</u>

46. Chaput J.-P., M. Weippert, T.V. Barreira, S.T. Broyles, M. Fogelholm, G. Hu, R. Kuriyan, A. Kurpad, E.V. Lambert, C. Maher, J. Maia, V. Matsudo, T. Olds, V. Onywera, O.L. Sarmiento, M. Standage, M.S. Tremblay, C. Tudor-Locke, P. Zhao and P.T. Katzmarzyk. Are children like werewolves? Full moon and its association with sleep and activity behaviors in an international sample of children. *Frontiers in Pediatrics* 2016:4:24.

47. Dumuid, D., T.S. Olds, L.K. Lewis, and C. Maher. Does home equipment contribute to socioeconomic gradients in Australian children's physical activity, sedentary time and screen time? *BMC Public Health* 2016;16(1): 736.
48. Ferrari G., V. Matsudo, T.V. Barreira, C. Tudor-Locke, Mire, P.T. Katzmarzyk and M. Fisberg. Correlates of moderate-to-vigorous physical activity in Brazilian children. *Journal of Physical Activity and Health* 2016;13:1132-1145.
49. Harrington D.M., S.T. Broyles, J.-P. Chaput, M. Fogelholm, F. Gillison, G. Hu, R. Kuriyan, A. Kurpad, E.V. Lambert, A.G. LeBlanc, C. Maher, J. Maia, V. Matsudo, T. Olds, V.O. Onywera, O.L. Sarmiento, M. Standage, M.S. Tremblay, C. Tudor-Locke, P. Zhao, T.S. Church and P.T. Katzmarzyk for the ISCOLE Research Group. Household level correlates of children's physical activity levels in and across twelve countries. *Obesity* 2016;24:2150-2157.
50. Katzmarzyk P.T., S.T. Broyles, C.M. Champagne, J.-P. Chaput, M. Fogelholm, G. Hu, R. Kuriyan, A. Kurpad, E.V. Lambert, J. Maia, V. Matsudo, T.S. Olds, V. Onywera, O.L. Sarmiento, M. Standage, M.S. Tremblay, C. Tudor-Locke and P. Zhao for the ISCOLE Research Group. Relationship between soft drink consumption and obesity in 9-11 year old children in a multi-national study. *Nutrients* 2016;8:770.
51. LeBlanc A.G., C. Boyer, M.M. Borghese, J.-P. Chaput, G. Leduc, M.S. Tremblay and P.E. Longmuir. Canadian physical activity and screen time guidelines: Do children know? *Health Behavior and Policy Review* 2016;3(5):444-454.
52. Lewis L.K., C. Maher, K. Belanger, M.S. Tremblay, J.-P. Chaput and T. Olds. At the mercy of the Gods: Associations between weather, physical activity and sedentary time in children. *Pediatric Exercise Science* 2016; 28:152-163.
53. Lewis L., C. Maher, P.T. Katzmarzyk and T.S. Olds. Individual and school-level socio-economic gradients in physical activity in Australian school children. *Journal of School Health* 2016;86:105-112.
54. Lim J., J.M. Schuna, Jr., M.A. Busa, B.R. Umberger, P.T. Katzmarzyk, R.E. Van Emmerik and C. Tudor-Locke. Allometrically scaled children's clinical and free-living ambulatory behaviour. *Medicine and Science in Sports and Exercise* 2016;48:2407-2416.
55. Lizotte C., R. Larouche, A.G. LeBlanc, P.E. Longmuir, M.S. Tremblay and J.-P. Chaput. Investigation of new correlates of physical literacy in children. *Health Behavior and Policy Review* 2016;3(2):110-122.
56. Maher C., L. Lewis, P.T. Katzmarzyk, D. Dumuid and T. Olds. The associations between physical activity, sedentary behaviour and academic performance. *Journal of Science and Medicine in Sport* 2016; 19:1004-1009.
57. Matsudo V., G. Ferrari, T.L. Araujo, L.C. Oliveira, E. Mire, T.V. Barreira, C. Tudor-Locke and P.T. Katzmarzyk. Socioeconomic status indicators, physical activity, and overweight/obesity in Brazilian children. *Revista Paulista de Pediatria* 2016; 34:162-170.
58. Muthuri S.K., J.L M. Wachira, V.O. Onywera and M.S. Tremblay. Associations between parental perceptions of the neighbourhood environment and childhood physical activity: results from ISCOLE-Kenya. *Journal of Physical Activity and Health*; 2016;13:333-343.
59. Muthuri S.K., L-J.M. Wachira, V.O. Onywera, M.S. Tremblay, J.-P. Chaput, M. Fogelholm, G. Hu, R. Kuriyan, A. Kurpad, E.V. Lambert, C. Maher, J. Maia, V. Matsudo, T. Olds, O.L. Sarmiento, M. Standage, C. Tudor-Locke, P. Zhao, T.S. Church and P.T. Katzmarzyk. Relationships between parental education and

overweight with childhood overweight and physical activity in 9-11 year old children: Results from a 12-country study. *PLoS One* 2016;11(8): e0147746.
60. Pereira S., A. Borges, T.N. Gomes, D. Santos, M. Souza, F.K. Dos Santos, R.N. Chaves, T.V. Barreira, D. Hedeker, P.T. Katzmarzyk and J.A.R. Maia. Correlates of children's compliance with moderate-to-vigorous physical activity recommendations: a multilevel analysis. *Scandinavian Journal of Medicine & Science in Sports* 2016; (8):842-851.
61. Roman-Viñas B., J.-P. Chaput, P.T. Katzmarzyk, M. Fogelholm, E.V. Lambert, C. Maher, J. Maia, T. Olds, V. Onywera, O.L. Sarmiento, M. Standage, C. Tudor-Locke and M.S. Tremblay MS for the ISCOLE Research Group. Proportion of children meeting recommendations for 24-hour movement guidelines and associations with adiposity in a 12-country study. *International Journal of Behavioural Nutrition and Physical Activity* 2016;13(1):123.
62. Uys M., S. T. Broyles, C. E. Draper, S. Hendricks, D. Rae, N. Naidoo, P.K. Katzmarzyk, and E.V. Lambert. Perceived and objective neighbourhood support for outside of school physical activity in South African children. *BMC Public Health* 2016;16:462.
63. Wilkie H., M. Standage, F. Gillison, S. Cumming and P.T. Katzmarzyk. Multiple lifestyle behaviours and overweight and obesity among 9-11 year old children: Results from the UK site of the International Study of Childhood Obesity, Lifestyle and the Environment. *BMJ Open* 2016;6:e010677
64. Zhao P., E. Liu, Y. Qiao, P.T. Katzmarzyk, J.-P. Chaput, M. Fogelholm, W.D. Johnson, R. Kuriyan, A. Kurpad, E.V. Lambert, C. Maher, J.A.R. Maia, V. Matsudo, T. Olds, V. Onywera, O.L. Sarmiento, M. Standage, M.S. Tremblay, C. Tudor-Locke and G. Hu for the ISCOLE Research Group. Maternal gestational diabetes and childhood obesity at 9-11 years old in 12 countries. *Diabetologia* 2016;59:2339-2348.

2017

65. Borghese M.M., M.S. Tremblay, G. Leduc, C. Boyer, P. Belanger, A.G. LeBlanc, C. Francis and J.-P. Chaput. Comparison of ActiGraph GT3X+ and Actical accelerometer data in 9-11 year old Canadian children. *J Sports Sci,* 2017; 35(6):517-524.
66. Chaput J.P., P.T. Katzmarzyk, J.D. Barnes, M. Fogelholm, G. Hu, R. Kuriyan, A. Kurpad, E.V. Lambert, C. Maher, J. Maia, V. Matsudo, T. Olds, V. Onywera, O.L. Sarmiento, M. Standage, C. Tudor-Locke, P. Zhao and M.S. Tremblay for the ISCOLE Research Group. Mid-upper arm circumference as a screening tool for identifying children with obesity: A 12-country study. *Pediatric Obesity* 2017 Dec;12(6):439-445.
67. Dumuid D., T.S. Olds, L. Lewis and C. Maher. Academic performance and lifestyle behaviours in Australian school children: A cluster analysis. *Health Education and Behavior* 2017;44(6):918-927.
68. Dumuid D., T.S. Olds, L. Lewis, J.A. Martin-Fernandez, P.T. Katzmarzyk, T.V. Barreira, S.T. Broyles, J.-P. Chaput, M. Fogelholm, G. Hu, Kuriyan, A. Kurpad, E.V. Lambert, J. Maia, V. Matsudo, V. Onywera, O.L. Sarmiento, M. Standage, M.S. Tremblay, C. Tudor-Locke, P. Zhao, F. Gillison and C. Maher for the ISCOLE Research Group. Health-related quality of life and lifestyle behaviour clusters in school-aged children from 12 countries. *Journal of Pediatrics* 2017;183:178-183.
69. Ferrari G., T.L. Araújo, L.C. Oliveira, V. Matsudo, E. Mire, T. Barreira, C. Tudor-Locke and P.T. Katzmarzyk. Accelerometer-determined peak cadence and weight

status in children from São Caetano do Sul, Brazil. *Ciência & Saúde Coletiva* 2017;22:3689-3698.

70. Ferrari G., V. Matsudo, P.T. Katzmarzyk and M. Fisberg. Prevalence and factors associated with body mass index in children aged 9-11 years. *Jornal de Pediatria* 2017;93(6):601-609.

71. Gillison F., S.P Cumming, M. Standage, C. Barnaby and P.T. Katzmarzyk. Assessing the impact of adjusting for maturity in children's weight status classification in a cohort of UK children. *BMJ Open* 2017;7:e015769.

72. Gillison F., M. Standage, S. Cumming, J. Zakrzewski-Fruer, P.C. Rouse and P.T. Katzmarzyk. Does parental support moderate the effect of children's motivation and self-efficacy on physical activity and sedentary behaviour? *Psychology of Sport and Exercise* 2017;32:153-161.

73. Gomes T.N., D. Hedeker, F.K. Dos Santos, M. Souza, D. Santos, S. Pereira, P.T. Katzmarzyk and J. Maia. Relationship between sedentariness and moderate-to-vigorous physical activity in youth: A multivariate multilevel study. *International Journal of Environmental Research and Public Health* 2017;14(2):148.

74. Gomes T.N., P.T Katzmarzyk, D. Hedeker, M. Fogelholm, M. Standage, V. Onywera, E.V. Lambert, M.S. Tremblay, J.-P. Chaput, C. Tudor-Locke, O. Sarmiento, V. Matsudo, A. Kurpard, R. Kuriyan, P. Zhao, G. Hu, T. Olds, C. Maher and J. Maia for the ISCOLE Research Group. Correlates of compliance with recommended levels of physical activity in children. *Scientific Reports* 2017;7:16507.

75. Manyanga T., T.V. Barreira, J.-P. Chaput, M. Fogelholm, G. Hu, P.T. Katzmarzyk. R. Kuriyan, A. Kurpad, E.V. Lambert, C. Maher, J. Maia, V. Matsudo, T. Olds, V. Onywera, O.L. Sarmiento, M. Standage, M.S. Tremblay. C. Tudor-Locke and P. Zhao. Socioeconomic status and dietary patterns in children from around the world: different associations by levels of country human development? *BMC Public Health* 2017:17:457.

76. Oliveira L.C., G. Ferrari, T.L. Araujo and V. Matsudo. Overweight, obesity, steps and moderate to vigorous physical activity in children. *Review Saude Publica* 2017;51(0):38.

77. Sampasa-Kanyinga H., M. Standage, M.S. Tremblay, P.T. Katzmarzyk, G. Hu, R. Kuriyan, C. Maher, J. Maia, T. Olds, O.L. Sarmiento, C. Tudor-Locke and J.-P. Chaput. Associations between meeting combinations of 24-h movement guidelines and health-related quality of life in children from 12 countries. *Public Health* 2017;153:16-24.

78. Sullivan S., S.T. Broyles, T.V. Barreira, J.P. Chaput, M. Fogelholm, G. Hu, W.D. Johnson, R. Kuriyan, A. Kurpad, E.V. Lambert, C. Maher, J. Maia, V. Matsudo, T. Olds,V. Onywera, O.L. Sarmiento, M. Standage, M.S. Tremblay, C. Tudor-Locke, P. Zhao and P.T. Katzmarzyk for the ISCOLE Research Group. Associations of neighborhood social environment attributes and physical activity among 9-11 year old children from 12 countries. *Health and Place* 2017;46:183-191.

79. Qiao Y., T. Zhang, H. Liu, P.T. Katzmarzyk, J.P. Chaput, M. Fogelholm, W.D. Johnson, R. Kuriyan, A. Kurpad, E.V. Lambert, C. Maher, J. Maia, V. Matsudo, T. Olds, V. Onywera, O.L. Sarmiento, M. Standage, M.S. Tremblay, C. Tudor-Locke, T.S. Church, P. Zhao and G. Hu. Joint association of birth weight and physical

activity/sedentary behavior with obesity in children ages 9-11 years from 12 countries. *Obesity* 2017;25:1091-1097.

<u>2018</u>

80. Chaput J.P., J.D. Barnes, M.S. Tremblay, M. Fogelholm, G. Hu, E.V. Lambert, C. Maher, J. Maia, T. Olds, V. Onywera, O.L. Sarmiento, M. Standage, C. Tudor-Locke and P.T. Katzmarzyk for the ISCOLE Research Group. Inequality in physical activity, sedentary behavior, sleep duration, and risk of obesity in children: A 12-country study. *Obesity Science and Practice* 2018; 4:(3):229-237
81. Chaput J.-P., J.D. Barnes, M.S. Tremblay, M. Fogelholm, G. Hu, E.V. Lambert, C. Maher, J. Maia, T. Olds, V. Onywera, O.L. Sarmiento, M. Standage, C. Tudor-Locke and P.T. Katzmarzyk for the ISCOLE Research Group. Thresholds of physical activity associated with obesity by level of sedentary behavior in children. *Pediatric Obesity* 2018;13(7):450-457.
82. Chaput J.-P., M.S. Tremblay, P.T. Katzmarzyk, M. Fogelholm, G. Hu, C. Maher, J. Maia, T. Olds, V. Onywera, M. Standage, C. Tudor-Locke and H. Sampasa-Kanying for the ISCOLE Research Group. Sleep patterns and sugar-sweetened beverage consumption among children from around the world. *Public Health Nutrition* 2018:21(13):2385-2393.
83. Chaput J.-P., M.S. Tremblay, P.T. Katzmarzyk, M. Fogelholm, V. Mikkilä, G. Hu, E.V. Lambert, C. Maher, J. Maia, T. Olds, V. Onywera, O.L. Sarmiento, M. Standage, C. Tudor-Locke and A.G. LeBlanc for the ISCOLE Research Group. *Journal of Public Health* 2018;40:e493-e501.
84. Dumuid D., C. Maher, L.K. Lewis, T.E. Stanford, J.A.M. Fernández, J. Ratcliffee, P.T. Katzmarzyk, T.V. Barreira, J.-P. Chaput, M. Fogelholm, G. Hu, J, Maia, O.L. Sarmiento, M. Standage, M.S Tremblay C. Tudor-Locke and T. Olds. Human development index, children's health-related quality of life and movement behaviors: a compositional data analysis. *Quality of Life Research* 2018;27(6):1473-1482.
85. Dumuid D., T.S. Olds, L.K. Lewis, J.A. Martin-Fernandez, P.T. Katzmarzyk, T.V. Barreira, S. Broyles, J.-P. Chaput, M. Fogelholm, G. Hu, R. Kuriyan, A. Kurpad, E.V. Lambert, J. Maia, V. Matsudo, V. Onywera, O.L. Sarmiento, M. Standage, M.S. Tremblay, C. Tudor-Locke, P. Zhao, F. Gillison, and C. Maher for the ISCOLE Research Group. The adiposity of children is associated with their lifestyle behaviours: a cluster analysis of school-aged children from 12 nations. *Pediatric Obesity* 2018;13(2):111-119.
86. Dumuid D., T.E. Stanford, J.A. Martin-Fernández, Z. Pedisic, C. Maher, L.K. Lewis, K. Hron, P.T. Katzmarzyk, J.-P. Chaput, M. Fogelholm, G. Hu, E.V. Lambert, J. Maia, O.L. Sarmiento, M. Standage, T.V. Barreira, S.T. Broyles, C. Tudor-Locke, M.S. Tremblay and T Olds. Compositional data analysis for physical activity, sedentary time and sleep research. *Statistical Methods in Medical Research* 2018;27:3726-3738.
87. Dumuid D., T.E. Stanford, Z. Pedišić, C. Maher, L.K. Lewis, J.A.M. Fernández, P.T. Katzmarzyk, J.-P. Chaput, M. Fogelholm, M. Standage, M.S. Tremblay and T. Olds. Adiposity and the isotemporal substitution of physical activity, sedentary time and sleep among school-aged children: A compositional data analysis approach. *BMC Public Health* 2018;18(1):311.
88. Katzmarzyk P.T., S.T. Broyles, J.-P. Chaput, M. Fogelholm, G. Hu, E.V. Lambert, C. Maher, J. Maia, T. Olds, V. Onywera, O.L. Sarmiento, M. Standage, M.S. Tremblay,

and C. Tudor-Locke for the ISCOLE Research Group. Sources of variability in childhood obesity indicators and related behaviors. *International Journal of Obesity and Related Disorders* 2018;42:108-110.

89. Lin Y., M.S. Tremblay, P.T. Katzmarzyk, M. Fogelholm, G. Hu, E.V. Lambert, C. Maher, J. Maia, T. Olds, O.L. Sarmiento, M. Standage, C. Tudor-Locke and J.-P. Chaput for the ISCOLE Research Group. Temporal and bi-directional associations between sleep duration and physical activity/sedentary time in children: an international comparison. *Preventive Medicine* 2018;111:436-441.

90. Manyanga T., J.D. Barnes, M.S. Tremblay, P.T. Katzmarzyk, S.T. Broyles, T.V. Barreira, M. Fogelholm, G. Hu, C. Maher, J. Maia, T. Olds, O.L. Sarmiento, M. Standage, C. Tudor-Locke and J.-P. Chaput for the ISCOLE Research Group. No evidence for an epidemiological transition in sleep patterns among children: a 12-country study. *Sleep Health* 2018;4:87-95.

91. Olds T., I. Sanders, C. Maher, F. Fraysse, L. Bell and E. Leslie. Does compliance with healthy lifestyle behaviours cluster within individuals in Australian primary school-aged children? *Child: Care, Health and Development* 2018;44(1):117-123.

92. Silva D.A.S., J.-P. Chaput, P.T. Katzmarzyk, M. Fogelholm, G. Hu, C. Maher, T. Olds, V. Onywera, O.L. Sarmiento, M. Standage, C. Tudor-Locke and M.S. Tremblay. Physical education classes, physical activity, and sedentary behavior in children. *Medicine and Science in Sports and Exercise* 2018;50(5):995-1004.

93. Wachira L.-J, S.K. Muthuri, S.A. Ochola, Onywera, V.O. and M.S. Tremblay. Screen-based sedentary behaviour and adiposity among school children: Results from International Study of Childhood Obesity, Lifestyle and the Environment (ISCOLE) – Kenya. *PLoS One* 2018;13(6):e0199790.

94. Wilkie H.J., M. Standage, F.B. Gillison, S.P. Cumming and P.T. Katzmarzyk. Correlates of intensity-specific physical activity in 9-11 year old children: a multilevel analysis of UK data from the International Study of Childhood Obesity, Lifestyle and the Environment. *BMJ Open* 2018;8:e018373.

2019

95. Thivel D., M.S. Tremblay, P.T. Katzmarzyk, M. Fogelholm, Vera Mikkilä, G. Hu, E.V. Lambert, C. Maher, J. Maia, T. Olds, V. Onywera, O.L. Sarmiento, M. Standage, C. Tudor-Locke and J.-P. Chaput for the ISCOLE Research Group. Associations between meeting combinations of 24-hour movement recommendations and dietary patterns of children: A 12-country study. *Preventive Medicine* 2019;118:159-165.

96. Larouche R., E.F. Mire, K. Belanger, T.V. Barreira, J.-P. Chaput, M. Fogelholm, G. Hu, E.V. Lambert, C. Maher, J. Maia, T. Olds, V. Onywera, O.L. Sarmiento, M. Standage, C. Tudor-Locke, P.T. Katzmarzyk and M.S. Tremblay for the ISCOLE Research Group Relationships between outdoor time, physical activity, sedentary time and body mass index in children: A 12-country study. *Pediatric Exercise Science* 2019;31:118-129.

97. Ferrari G., C. Pires, D. Sole, V. Matsudo, P.T. Katzmarzyk and M. Fisberg. Factors associated with objectively measured total sedentary time and screen time in children aged 9-11 years. *Jornal de Pediatrica* (Rio J). 2019; 95:94-105.

98. Reyes C.A.T., O.L. Sarmiento, A. Bravo-Balado, S.A. González, M.A. Bolívar, P. Lemoine, J.D. Meisel, C. Grijalba and P.T Katzmarzyk. Active streets for children: The case of the ciclovía of Bogotá. *PLOS One* 2019;In Press: Accepted for Publication on November 7, 2018.

99. Li N., P. Zhao, C. Diao, Y. Qiao, P.T. Katzmarzyk, J.-P. Chaput, M. Fogelholm, R. Kuriyan, A. Kurpad, E.V. Lambert, C. Maher, J. Maia, V. Matsudo, T. Olds, V. Onywera, O.L. Sarmiento, M. Standage, M.S. Tremblay, C. Tudor-Locke and G..Hu for the ISCOLE Research Group. Joint associations between weekday and weekend physical activity or sedentary time and childhood obesity. *International Journal of Obesity and Related Metabolic Disorders* 2019;43:691-700.
100. Jalo E., H. Konttinen, H. Vepsäläinen, J.-P. Chaput, G. Hu, C. Maher, J. Maia, O.L. Sarmiento, M. Standage, C. Tudor-Locke, P.T. Katzmarzyk and M. Fogelholm. Emotional eating, health behaviours, and obesity in children: A 12-country cross-sectional study. *Nutrients* 2019;11(2):pii:E351.
101. Zakrzewski-Fruer J., F.B. Gillison, P.T. Katzmarzyk, E.F. Mire, S.T. Broyles, C.M. Champagne, J.-P. Chaput, K.D. Denstel, M. Fogelholm, G. Hu, E.V. Lambert, C. Maher, J. Maia, T. Olds, V. Onywera, O.L. Sarmiento, M.S. Tremblay, C. Tudor-Locke and M. Standage for the ISCOLE Research Group. Association between breakfast frequency and physical activity and sedentary time: A cross-sectional study in children from 12 countries. *BMC Public Health* 2019;19:222.
102. Barreira T.V., S.T. Broyles, C. Tudor-Locke, J.-P. Chaput, , M. Fogelholm, G. Hu, R. Kuriyan, E.V. Lambert, C. Maher, J. Maia, T. Olds, V. Onywera, O.L. Sarmiento, M. Standage, M.S. Tremblay and P.T. Katzmarzyk for the ISCOLE Research Group. Epidemiological transition in physical activity and sedentary time in children. *Journal of Physical Activity and Health* 2019; In Press: Accepted for Publication on February 22, 2019.
